# A first-passage approach to diffusion-influenced reversible binding and its insights into nanoscale signaling at the presynapse

**DOI:** 10.1038/s41598-021-84340-4

**Published:** 2021-03-08

**Authors:** Maria Reva, David A. DiGregorio, Denis S. Grebenkov

**Affiliations:** 1 Unit of Synapse and Circuit Dynamics, CNRS UMR 3571, Institut Pasteur, Paris, France; 2 ED3C, Sorbonne University, Paris, France; 3 Laboratoire de Physique de la Matière Condensée (UMR 7643), CNRS – Ecole Polytechnique, IP Paris, 91128 Palaiseau, France

**Keywords:** Biophysics, Cell signalling, Molecular neuroscience, Transporters in the nervous system, Statistical physics, thermodynamics and nonlinear dynamics, Chemical physics

## Abstract

Synaptic transmission between neurons is governed by a cascade of stochastic calcium ion reaction–diffusion events within nerve terminals leading to vesicular release of neurotransmitter. Since experimental measurements of such systems are challenging due to their nanometer and sub-millisecond scale, numerical simulations remain the principal tool for studying calcium-dependent neurotransmitter release driven by electrical impulses, despite the limitations of time-consuming calculations. In this paper, we develop an analytical solution to rapidly explore dynamical stochastic reaction–diffusion problems based on first-passage times. This is the first analytical model that accounts simultaneously for relevant statistical features of calcium ion diffusion, buffering, and its binding/unbinding reaction with a calcium sensor for synaptic vesicle fusion. In particular, unbinding kinetics are shown to have a major impact on submillisecond sensor occupancy probability and therefore cannot be neglected. Using Monte Carlo simulations we validated our analytical solution for instantaneous calcium influx and that through voltage-gated calcium channels. We present a fast and rigorous analytical tool that permits a systematic exploration of the influence of various biophysical parameters on molecular interactions within cells, and which can serve as a building block for more general cell signaling simulators.

## Introduction

Intracellular transport of molecules is crucial for the normal function and growth of living cells^1^. Many intracellular signaling cascades, such as those mediated by calcium ions ($Ca^{2+}$), are generated by biochemical reactions and diffusion, which are often hard to accurately measure on the submillisecond and submicron temporal and spatial scales^2^. Mathematical modeling can be used to interpret and predict features of intracellular signaling that are not yet directly observable. One particularly interesting signaling process is the ability of neurons in the brain to communicate with each other by transforming electrical into chemical signals and then back to electrical signals at specialized junctions called synapses. Electrical impulses, or action potentials (APs), recruit voltage-gated calcium channels (VGCC) that mediate $Ca^{2+}$ fluxes across the membrane followed by diffusion and binding to buffer molecules throughout the presynaptic terminal. When free $Ca^{2+}$ encounter and bind to target $Ca^{2+}$sensor proteins, which are tethered to neurotransmitter containing synaptic vesicles (SVs), SV fusion with the plasma membrane is triggered and neurotransmitter is released into the synaptic cleft. The neurotransmitter molecules diffuse within the synaptic cleft to bind neurotransmitter-gated ion channels on the postsynaptic cell and then initiate an electrical signal. $Ca^{2+}$ entry and diffusion to the $Ca^{2+}$sensor are thought to occur within tens of nanometers on a sub-millisecond timescale^3,4^. Intracellular $Ca^{2+}$-binding proteins can act as buffers, which play an important role in shaping the spatial and temporal dynamics of intracellular $Ca^{2+}$ concentration ([$Ca^{2+}$]) gradients (unbound or free ions)^5^. These dynamic concentration gradients in turn determine the time course of the $Ca^{2+}$ occupancy of the sensor. Therefore, numerical simulations of chemical reactions and $Ca^{2+}$ diffusion have been essential for understanding the spatial-temporal dynamics of the [$Ca^{2+}$] driving synaptic vesicle fusion^3,4^.

An explicit analytical solution of the $Ca^{2+}$ reaction–diffusion equations describing the coupling between transient $Ca^{2+}$ fluxes and the occupancy of the $Ca^{2+}$ sensor for SV fusion is not possible. Therefore, deterministic^4,6^ and stochastic^7,8^ simulations have been workhorses to study this problem. However, both strategies are time-consuming and suffer from inaccuracies under physiologically relevant parameter regimes. The finite element methods^4^ do not account for the stochastic opening of VGCCs or fluctuations in $Ca^{2+}$ flux, which should be simulated explicitly in order to accurately predict vesicle fusion probability^4,9^. This motivates the use of Monte Carlo methods that can generally be divided into two groups: particle-based and lattice-based. In the particle-based methods each particle is treated individually, making computation time prohibitively expensive when the concentrations of particles are high and/or there are numerous species (e.g. a large number of ions). The lattice-based methods divide space into voxels and treat diffusants as concentrations rather than individual particles^10,11^. This approach can speed up the simulations, but at the price of reduced spatial and temporal resolution. Moreover, MC techniques suffer from inaccurate simulations of statistically rare events, despite recent advances in sampling methods^12^. In this context, analytical solutions of simplified models can provide new insights and intuition into complex systems, as well as much faster simulation speeds. In particular, analytical solutions allow for rapid exploration of a vast parameter space to reveal relevant spatial and temporal scales of specific parameter combinations.

**Figure 1:**
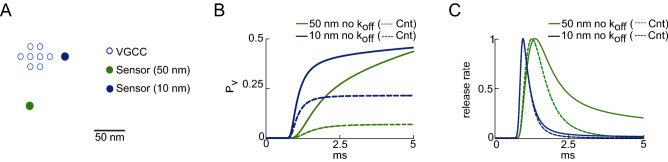
(**A**) Diagram of the active zone arrangement, with a cluster of VGCC (hollow circles) and two positions of SV sensors at 10 and 50 nm. (**B**) Vesicle release probability for two scenarios: control (with unbinding reaction,“Cnt”, dashed lines) and without unbinding reactions in the sensor kinetics (solid lines), and two CDs: 10 nm (blue) and 50 nm (green). (**C**) Release rates (corresponding to panel (**B**)), normalized to peak amplitude.

A well-established example of such an approach is the linearized buffer approximation (LBA^13^), which yields an approximate analytical solution of the related diffusion–reaction problem. This can be extended to multiple buffers and has provided important intuition about their impact on the spatial and temporal profile of intracellular $Ca^{2+}$, and the potential effect on the probability of SV fusion. However, this approach can only be applied under steady state conditions, and thus is not suitable for the brief and transient $Ca^{2+}$ influx driven by APs^14^. Another recent multi-scale approach is based on the narrow escape problem^15–17^ of searching for a hidden target by a single calcium ion. An analytical solution to this problem was found and then coupled with a Markovian jump process to model buffering and calcium influx^18^. Despite its advantages, this hybrid method does not account for the $Ca^{2+}$sensor’s binding and unbinding kinetics, which is of crucial importance for the vesicle release dynamics, as shown below (Fig. 1). In turn, recent first-passage approaches have been used to account for the finite backward rate constant of binding for multiple particles^17,19^, but do not consider competing binding partners for diffusants, which is necessary for biological realism and is the advantage of LBA. Finally, as all cells are known to have endogenous $Ca^{2+}$ buffers, a simulation approach that can account for diffusion and reaction with a target and competing binding reactions is essential to model calcium-dependent SV fusion.

Here we propose a probabilistic diffusion-influenced reversible calcium-binding model that overcomes the aforementioned deficiencies by considering the forward and backward binding rate constants of the $Ca^{2+}$sensor, as well as competing binding partners (endogenous fixed buffer (EFB) and mobile buffers). This novel analytical model simulates a point source $Ca^{2+}$ entry, reaction with buffers, diffusion and binding to a $Ca^{2+}$ sensor for SV fusion. The solution allows us to study the effect of binding reaction rate constants on the occupancy probability of the sensor by $Ca^{2+}$ at all temporal scales with much lower computational costs than any existing numerical alternatives (see below). We confirmed the necessity of taking into account the unbinding kinetics in the simulations of vesicle release probabilities. We also demonstrate the validity of the analytical solution by Monte Carlo simulations and study the effects of the sensor’s kinetics and geometrical properties of the synapse on the probability of the single site occupancy. Moreover, an extension to multiple calcium ions and its limitations are discussed. To our knowledge, this is the first analytical solution for a stochastic reaction–diffusion problem that accounts simultaneously for binding/unbinding kinetics of a single binding site sensor in the presence of competing buffer species, and accurately predicts target occupancies following stochastic influx from ion channels, as compared to particle-based Monte Carlo simulations. We also show how a cooperative, multiple independent binding site release sensor can be implemented analytically. This approach is therefore applicable to a wide range of biochemical processes within cells that operate via diffusion-influenced reactions.

## Results

### Impact of unbinding kinetics on the vesicle fusion probability and time course

Reversible first-order chemical reactions are described by forward ($k_{\mathrm{on}}$) and backward ($k_{\mathrm{off}}$) rate constants. However, in the case of $Ca^{2+}$ diffusion and binding to a $Ca^{2+}$ sensor for SV fusion, it has been argued that the first passage to the target is the dominant physical process influencing the probability of SV fusion over time, and thus approximations without $k_{\mathrm{off}}$ might be sufficient. We tested the importance of $Ca^{2+}$ sensor $k_{\mathrm{off}}$ for AP-evoked SV fusion by solving reaction–diffusion equations by a finite-element method (see “Methods” section). Spatio-temporal profiles of free [$Ca^{2+}$] were simulated for sensor distances of 10 and 50 nm from the perimeter of a VGCC cluster (perimeter model^4,20^, see Fig. 1A) in the presence of $Ca^{2+}$ buffers (ATP or endogenous fixed buffers).

We modeled the probability of SV fusion within the five binding site kinetic model of the $Ca^{2+}$ sensor^21^ (see “Methods” section) and compared it to a model in which all $k_{\mathrm{off}}$s were set to zero. For sensor-to-channel distances (coupling distance, CD) as short as 10 nm, the time course of SV release within the first millisecond is hardly different with and without a $k_{\mathrm{off}}$ (Fig. 1C, blue lines), while the release probability is increased by 2.4 times (Fig. 1B, blue lines). However, in the case of 50 nm CD (which is physiological at some synapses^20,22^), setting the $k_{\mathrm{off}}$ to zero increased the vesicle release probability by 7-fold (Fig. 1B, green lines) and increased the half-width of the time course of fusion probability by 61% (Fig. 1C, green lines). These simulations show that for the shortest CDs, first passage time models that only consider $k_{\mathrm{on}}$ could qualitatively reproduce the time evolution in sensor occupancy, but not the final probability of SV fusion. However, for longer CDs, both the time course and fusion probability were altered in the absence of unbinding. Thus, a reversible $Ca^{2+}$ binding reaction (finite $k_{\mathrm{off}}$) must be considered for such simulations, particularly since the estimated coupling distances range from 10 nm to 100 nm across synapses^20^. Moreover, by solving analytically the governing reaction–diffusion equations, we would provide an efficient framework for studying and modeling the dynamics of molecular diffusion and binding to a partner. In the following sections, we investigate analytical solutions that describe, specifically, $Ca^{2+}$ diffusion and consider explicitly the binding and unbinding kinetics of the sensor.

### Analytical model of $Ca^{2+}$ reaction–diffusion

**Figure 2:**
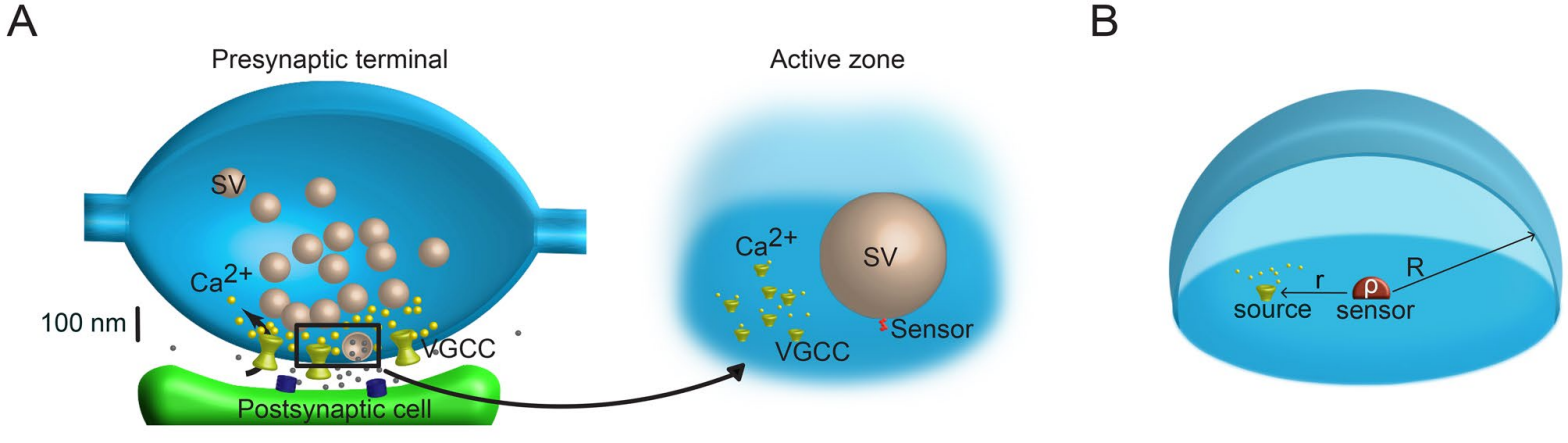
(**A**) Schematic diagram of an axonal bouton (presynaptic terminal) containing a release site (active zone). Inset: Idealized active zone scheme showing VGCC clusters and their tens of nanometers proximity to the $Ca^{2+}$ target sensor for SV fusion. (**B**) Geometric representation of the simulation domain. The simulation compartment is a reflecting half-sphere of radius *R*, target is a partially absorbing half-sphere of radius $\rho$, the point source of $Ca^{2+}$ entry is located on the membrane (horizontal surface) at the coupling distance (CD), $r-\rho$, from the sensor (target).

Understanding the lifetime of calcium ions on single binding sites of a SV fusion sensor and the influence of competing buffers is essential for studying the nanoscale signaling driving neurotransmitter release at synapses. As a first approximation of this process, we developed an analytical model of $Ca^{2+}$ diffusion based on the first passage time concept, in which the target $Ca^{2+}$ sensor binding occupancy was modeled as a first-order reaction with reversible kinetics (both $k_{\mathrm{on}}$ and $k_{\mathrm{off}}$) . This probabilistic diffusion-influenced reversible calcium-binding model is described in detail in the “Methods” section (see also Fig. 2). In brief, we placed a single $Ca^{2+}$ sensor, with the values of $k_{\mathrm{on}}$ and $k_{\mathrm{off}}$ taken from models predicting experimental data^21^, at the center of the circular surface of a half-sphere. We assumed an unlimited binding capacity of the sensor that permits each $Ca^{2+}$ binding event to occur independently. The hindering of diffusion by synaptic vesicles was not considered, since it was shown not to influence sensor occupancy^14^. The dynamics of $Ca^{2+}$ ions was modeled as switching diffusion between free and buffer-bound states^23,24^. In summary, our model has the following parameters: the size of the sensor $\rho$, the distance between the origin of the simulation domain and calcium channel *r*, the radius of the simulation domain *R* (see Fig. 2B), $k_{\mathrm{on}}$ and $k_{\mathrm{off}}$ of the $Ca^{2+}$ sensor, the exchange rates $k_{0i}$ and $k_{i0}$ (product of concentration and forward rate constant of the buffer) for binding/unbinding to *i*-th buffer, and diffusion coefficients of free $Ca^{2+}$ ($D_0$) and those bound to buffers ($D_i$—diffusion coefficient of *i*-th buffer), see Table 1.

In order to derive a set of equations describing the solution, we took a two-step approach. First, we found the probability distribution of the first-passage time of a $Ca^{2+}$ ion to a simplified (single binding site) sensor in the presence of competing binding partners for a single $Ca^{2+}$ entering the bouton. Second, a renewal technique^25^ allowed us to incorporate unbinding kinetics on the sensor and to relate the distribution of the first-binding time to the occupancy probability, *P*(*t*, *r*), for a single $Ca^{2+}$ ion started at some position *r*, to be on the sensor at time *t* (see “Methods” section, Eqs. (18)–(24)). While our first-passage approach applies to any number of co-existing buffers, we focused on two cases of no buffer and single buffer, for which explicit analytical formulas for *P*(*t*, *r*) were provided. We explored the accuracy and assumptions of these formulas using Monte Carlo simulations (see “Methods” section). Finally, we extended the analytical solution to account for multiple binding sites of the $Ca^{2+}$ sensor and for $Ca^{2+}$ fluxes elicited simultaneously or progressively through a single or multiple stochastic VGCCs. By solving the governing reaction–diffusion equations analytically, we provide an efficient framework for studying and modeling the dynamics of $Ca^{2+}$ diffusion and binding a molecular target, in particular AP driven $Ca^{2+}$-mediated SV fusion.

**Table 1:** Biophysical parameters of our diffusion–reaction model of the synaptic vesicle fusion sensor occupancy.

Parameter	Notation	Value	Unit	Reference
**Geometrical parameters**				
Simulation domain radius	*R*	300	${\mathrm {nm}}$	Adapted from Ref.^26^
Sensor’s radius	$\rho$	5	${\mathrm {nm}}$	Adapted from Ref.^27^
Coupling distance (CD)	$r - \rho$	15	${\mathrm {nm}}$	
Calcium diffusion coefficient	$D_0$	0.22	$\upmu {\mathrm{m}}^{2}\,{\mathrm{ms}}^{-1}$	^28^
***Ca***^**2+**^ **sensor**				
Forward rate constant	$k_{\mathrm{on}}$	$5\cdot 127$	${\text {mM}}^{-1}\, {\text {ms}}^{-1}$	^21^
Backward rate constant	$k_{\mathrm{off}}$	15.7	${\text {ms}}^{-1}$	^21^
**EFB** **(*i*** **= 1)**				
Diffusion coefficient	$D_1$	0	$\upmu {\mathrm{m}}^{2}\,{\mathrm{ms}}^{-1}$	
Backward rate constant	$k_{10}$	10	${\text {ms}}^{-1}$	
Forward rate constant	$k_{\mathrm{{on}},1}$	100	${\text {mM}}^{-1}\, {\text {ms}}^{-1}$	^29^
Total concentration	$c_1$	4	mM	^4^
Binding rate	$k_{01}$	400	${\text {ms}}^{-1}$	
**ATP buffer** **(*i* = 2)**				
Diffusion coefficient	$D_2$	0.2	$\upmu {\mathrm{m}}^{2}\,{\mathrm{ms}}^{-1}$	
Backward rate constant	$k_{20}$	10	${\text {ms}}^{-1}$	^13^
Forward rate constant	$k_{\mathrm{{on}},2}$	100	${\text {mM}}^{-1}\, {\text {ms}}^{-1}$	^13^
Total concentration	$c_2$	0.2	mM	^4^
Binding rate	$k_{02}$	20	${\text {ms}}^{-1}$	
**EGTA buffer** **(*i* = 3)**				
Diffusion coefficient	$D_3$	0.22	$\upmu {\mathrm{m}}^{2}\,{\mathrm{ms}}^{-1}$	^13^
Backward rate constant	$k_{30}$	0.000735	${\text {ms}}^{-1}$	^30^
Forward rate constant	$k_{\mathrm{{on}},3}$	10.5	${\text {mM}}^{-1}\, {\text {ms}}^{-1}$	^30^
Total concentration	$c_3$	10	mM	^4^
Binding rate	$k_{03}$	105	${\text {ms}}^{-1}$	

### Single ion occupancy probability for a single $Ca^{2+}$ binding site of the SV fusion sensor

**Figure 3:**
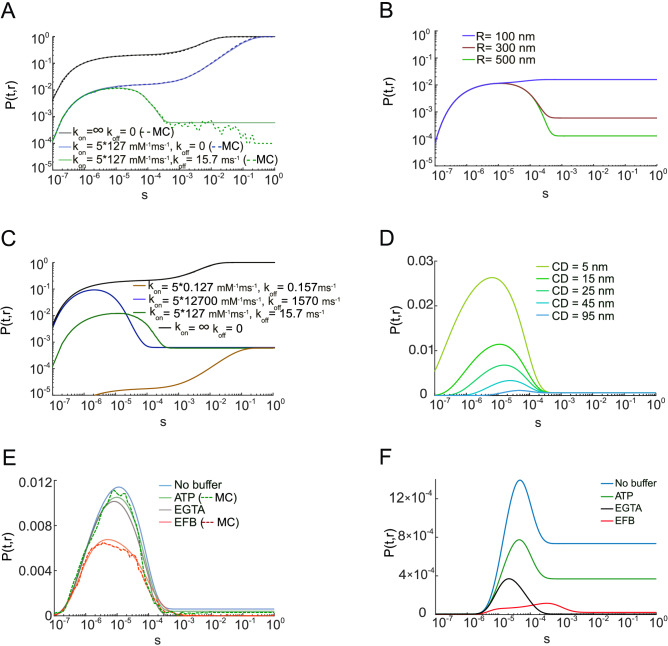
The occupancy probability *P*(*t*, *r*). (**A**) *P*(*t*, *r*) computed using an analytical solution (solid lines) and MC simulations (dashed lines) for the case of instant binding (black lines); in the presence of binding kinetics (blue lines); in the presence of both referent binding and unbinding kinetics (green lines). (**B**) Analytically computed *P*(*t*, *r*) for different sizes of the domain (*R*): 100 nm (blue), 300 nm (red) and 500 nm (green). (**C**) *P*(*t*, *r*) computed using an analytical solution for instant binding (black line), fast reaction rate constants (blue line), referent (green line) and slow reaction rate constants (brown line). (**D**) Analytically computed *P*(*t*, *r*) for CDs, $r-\rho$, varying from 5 to 95 nm. (**E**) *P*(*t*, *r*) computed using an analytical solution (solid lines) and MC simulations (dashed lines) in the absence (blue) or presence of one of the following buffers: ATP (green), EGTA (gray) and EFB (red), at the CD of 15 nm. (**F**) Similar to (**E**), but at the CD of 45 nm.

Using our analytical solution, it was possible to calculate the occupancy probability of a single binding site of the SV sensor by a single calcium ion, *P*(*t*, *r*), across seven orders of magnitude in time scales, from sub-microseconds to seconds, using different model parameters. For the idealized case of instantaneous binding and no unbinding ($k_{\mathrm{on}}= \infty$, $k_{\mathrm{off}}= 0$), any $Ca^{2+}$ ion that hits the sensor remains bound forever. As a consequence, *P*(*t*, *r*) is equal to the cumulative distribution function of the first passage time to the sensor. As expected, this probability monotonically increases with time and approaches 1 after one second (Fig. 3A, black solid line), consistent with a pure diffusion-limited reaction. The analytical solution is in excellent agreement with MC simulations, using the same model parameters (Fig. 3A, black dashed line). When using a finite forward rate constant ($k_{\mathrm{on}}= 5\cdot 127~{\text {mM}}^{-1}\, {\text {ms}}^{-1}$, $k_{\mathrm{off}}= 0$), the *P*(*t*, *r*) was reduced (Fig. 3A, blue solid line), in excellent agreement with MC simulations (Fig. 3A, blue dashed line). The use of both finite forward and backward rate constants generated a biphasic occupancy curve: the *P*(*t*, *r*) increased to a maximum value and followed by a decrease to a steady-state value, as expected physiologically. The curve's rising phase matched that of the curve when $k_{\mathrm{off}}$ was set to zero (Fig. 3A, green solid line), thereby delineating the time scale where only ion binding is dominant. MC simulations reproduced the analytical solutions (Fig. 3A, green dashed line), despite the inherent fluctuations due to a limited number of MC trials. It is worth mentioning that no $Ca^{2+}$ extrusion mechanism was considered here (see the “Methods” section). While this mechanism does not affect the early time scale behavior of the occupancy probability, active processes mediating $Ca^{2+}$ clearance can become relevant at long times of the order of seconds.

When increasing the size of the bouton (simulation volume), *R* = 500 nm, the peak of *P*(*t*, *r*) was not altered, but the steady-state *P*(*t*, *r*) was decreased ($1\cdot 10^{-3}$ for $R =300~{\mathrm {nm}}$, $3\cdot 10^{-4}$ for $R = 500~{\mathrm {nm}}$), consistent with alteration in the steady-state, volume-averaged [$Ca^{2+}$] (Fig. 3B). For smaller bouton sizes (*R* = 100 nm), the peak of *P*(*t*, *r*) was increased ($2\cdot 10^{-2}$). However, the rising phases were identical for all tested radii, suggesting that diffusion determines the initial time course of the dynamics of calcium ions accumulation on the SV sensor, provided that the bouton volume is much larger than the CD.

The probability *P*(*t*, *r*) was then computed for different pairs of forward and backward rate constants, each chosen such that equilibrium dissociation constant remains constant ($K_D= k_{\mathrm{on}}/k_{\mathrm{off}}\approx 40~ {\text {mM}}^{-1}$). In the three considered cases of fast ($k_{\mathrm{on}}= 5\cdot 12700~{\text {mM}}^{-1}\, {\text {ms}}^{-1}$, $k_{\mathrm{off}}= 1570~ {\text {ms}}^{-1}$), the reference ($k_{\mathrm{on}}= 5\cdot 127~{\text {mM}}^{-1}\, {\text {ms}}^{-1}$, $k_{\mathrm{off}}= 15.7~ {\text {ms}}^{-1}$), and slow ($k_{\mathrm{on}}= 5\cdot 0.127~{\text {mM}}^{-1}\, {\text {ms}}^{-1}$, $k_{\mathrm{off}}= 0.157~{\text {ms}}^{-1}$) rate constants, the *P*(*t*, *r*) reach the same equilibrium (Fig. 3C). However, within the first few hundreds of microseconds, faster rate constants enable a rapid capturing of $Ca^{2+}$ as well as faster unbinding, thus generating biphasic *P*(*t*, *r*) that is larger, briefer and earlier (Fig. 3C, blue line). Interestingly even with a forward rate-constant greater than $10^{10}$ $\hbox {M}^{-1}$ $\hbox {s}^{-1}$, the diffusion-limited case is not matched on the sub-microsecond time scale (Fig. 3C, black line) due to the fast $k_{\mathrm{off}}$. Thus, the $k_{\mathrm{off}}$ strongly influences target occupancy and the regime of diffusion-limited binding.

The distance between $Ca^{2+}$ sources (VGCCs) and the $Ca^{2+}$ sensor of SVs can vary across synapse types, influencing the kinetics and probability of SV fusion^3^. Therefore, we examined the effect of varying this VGCC-SV coupling distance in the range between 5 and 95 nm on *P*(*t*, *r*) (Fig. 3D). The peak of *P*(*t*, *r*) decreased from 0.027 at 5 nm to 0.001 at 95 nm. As expected from finite elements simulations^4,31^, longer CDs resulted in smaller peak $Ca^{2+}$-occupation probabilities and longer times to peak occupation (6.1 $\upmu$s at 5 nm to 47.5 $\upmu$s at 95 nm).

Finally, we explored the effect of various $Ca^{2+}$ buffers that critically shape the spatio-temporal profile of intracellular $Ca^{2+}$. We explored the effect of ATP, a naturally occurring low-affinity, fast and mobile endogenous calcium buffer^13^, non-specific low-affinity endogenous fixed buffers (EFB)^29^, and the mobile exogenous buffer EGTA (see Table 1). EGTA is a well-characterized buffer with a slow forward $Ca^{2+}$ binding rate constant that has been used to infer VGCC-SV CDs through competition with the $Ca^{2+}$ sensor, thus reproducing experimental inhibition of synaptic transmission, which was found to vary with CD^3,14^. Because of its slow $k_{\mathrm{on}}$, large concentrations of EGTA are needed to intercept $Ca^{2+}$ before they bind to the sensor for SV fusion (larger than 1 mM and up to 10 mM^14^). For particle-based simulations, this can be computationally prohibitive. The effect of all three buffers have been studied extensively, and thus have well-characterized binding rate constants^4^, see Table 1. Using our analytical approach we could rapidly calculate *P*(*t*, *r*) for a CD of 15 nm in the presence of either 0.2 mM ATP, or 10 mM EGTA. Both buffers only slightly decreased the peak amplitude of *P*(*t*, *r*) from 0.012 to 0.01 and shifted the time of its peak from 10 to 8.5 $\upmu$s. On the other hand, a high concentration of EFB (4 mM) had a more prominent effect, decreasing the peak probability of being bound to $7\cdot 10^{-3}$ and shifting its time to 4 $\upmu$s (Fig. 3E). These results are consistent with the lack of effect of ATP due to its low concentration and the lack of effect of EGTA due to its slow forward rate constant. At a CD of 45 nm, the *P*(*t*, *r*) peak was decreased from $1\cdot 10^{-3}$ to $3\cdot 10^{-4}$ (EGTA), $4\cdot 10^{-5}$ (EFB) and $6\cdot 10^{-3}$ (ATP); the time of peak was shifted from 4.7 to 2.1 $\upmu$s (EGTA), 9 $\upmu$s (EFB) and 4.5 $\upmu$s (ATP) (Fig. 3F). The steady-state $Ca^{2+}$ occupancy is dramatically reduced by the large concentration of the high-affinity buffer, EGTA. These differential effects of EGTA on the peak occupancy for CD of 15 and 45 nm, as well as on the steady-state occupancy, are very similar to previous analytical^13^, finite elements simulations^4,31^, and MC simulations^20^. The analytical solution was verified with MC simulations for ATP and EFBs (Fig. 3E), but not for EGTA as the large number of molecules associated with 10 mM EGTA was too time-consuming for MC simulations.

It was not possible to verify the analytical solution on Fig. 3F with our MC method due to the inability of the MC simulations to capture the rare binding events, even when the number of trials was increased to 50 000. These results illustrate the advantage of the analytical approach to provide an intuitive understanding of stochastic reaction and diffusion across a wide range of timescales and for large numbers of molecules, conditions that are prohibitive when using particle-based simulators.

### The temporal regime in which reaction–diffusion models must consider reversible binding with its target

**Figure 4:**
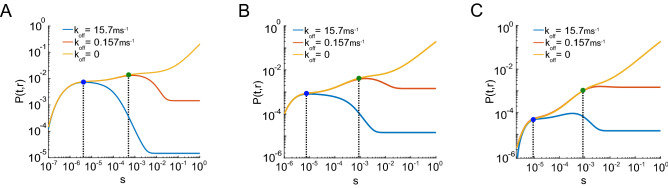
Influence of backward rate constant on *P*(*t*, *r*). (**A**) *P*(*t*, *r*) computed using analytical solution with fixed $k_{\mathrm{on}}= 5\cdot 127~{\text {mM}}^{-1}\, {\text {ms}}^{-1}$, and the following unbinding rates: $k_{\mathrm{off}}= 0$ (yellow line), $k_{\mathrm{off}}= 0.157~{\text {ms}}^{-1}$ (red line) and $k_{\mathrm{off}}=15.7~{\text {ms}}^{-1}$ (blue line), for the CD of 15 nm. The departure between *P*(*t*, *r*)s is depicted by dots and vertical dashed lines. Blue dot: $t_c \simeq 2.1~\upmu$s, green dot: $t_c \simeq 510~\upmu$s. (**B**) Similar to (**A**), for CD of 45 nm. Blue dot: $t_c \simeq 6.5~\upmu$s, green dot: $t_c \simeq 840~\upmu$s. (**C**) Similar to (**A**), for CD of 95 nm. Blue dot: $t_c \simeq 9.9~\upmu$s, green dot: $t_c \simeq 980~\upmu$s.

Equipped with an analytical solution, we reexamined the importance of $k_{\mathrm{off}}$ in dictating *P*(*r*, *t*). Figure 4 shows *P*(*r*, *t*) calculated for different $k_{\mathrm{off}}$s and different CDs: 15 nm (Fig. 4A), 45 nm (Fig. 4B) and 95 nm (Fig. 4C). The high temporal resolution of the simulations show that there is a characteristic time window $(0,t_c)$ at which the sensor occupancy is independent of the $k_{\mathrm{off}}$. The upper limit $t_c$ of this characteristic time window was defined as the time point when two *P*(*t*, *r*) curves for different unbinding kinetics start to deviate (blue and green dots, Fig. 4). This characteristic time increases as $k_{\mathrm{off}}$ decreases. For CDs less than 50 nm, the characteristic time window was less than 10 microseconds for physiological rate constants (Fig. 4A,B). The simplified first passage time approach confirms finite element simulations (Fig. 1) showing that backward rate constants in the physiological range can influence the $Ca^{2+}$ sensor’s occupancy for physiological source to target distances, and therefore must be modeled explicitly for accurate predictions of $Ca^{2+}$-dependent SV fusion.

### Sensor occupancy probability for multiple calcium ions

**Figure 5:**
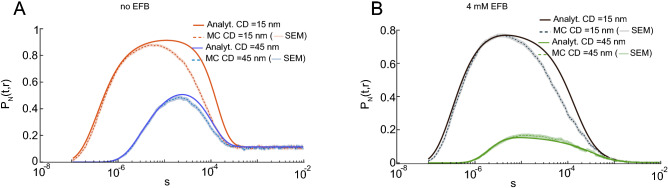
Occupancy probability, $P_N(t,r)$, for an instantaneous influx of many $Ca^{2+}$ ions ($N = 200$), computed using analytical method (solid lines) and MC simulations (dashed line; SEM: shaded region). (**A**) In the absence of fixed buffer, for CD of 15 nm (red lines) and CD of 45nm (blue lines). (**B**) Similarly in the presence of EFB (4 mM), CD of 15 nm (black lines) and CD of 45 nm (green lines).

Thus far, we considered the case when only a single $Ca^{2+}$ enters the presynaptic volume. We next explored the performance of our analytical solution for fluxes of multiple $Ca^{2+}$. During action potential-induced opening of a single VGCC, we estimate that approximately 200 $Ca^{2+}$ ions enter over a Gaussian-like time course (half-width  0.3 ms)^14,20^. Knowing *P*(*r*, *t*) for the single $Ca^{2+}$ ion, we used Eq. (25) to approximate the probability $P_N(r,t)$ that at least one $Ca^{2+}$ is bound to the sensor at time *t* following an instantaneous influx of *N* $Ca^{2+}$ ions (see “Methods” section). For an instantaneous flux of 200 ions, $P_N(t,r)$ was shown to increase to nearly 1 for a CD of 15 nm, in contrast to the low probabilities ($<0.01$) in the single-ion case. A similar peak was estimated using MC simulations, but the time course of analytical $P_N(t,r)$ was broader than that computed using MC simulations (Fig. 5A, dashed line; the shaded region is the standard error of the mean (SEM)). This difference between the analytical solution and the MC simulation was smaller for the longer CD of 45 nm (Fig. 5A, blue lines). This discrepancy shows a shortcoming of the analytical approximation when simulating multiple $Ca^{2+}$ ions. Because the analytical model considers an infinite number of binding sites, occupancy by one calcium ion does not occlude the binding of subsequent ions. However, this is not the case for real molecular interactions. Thus, the discrepancy between the MC and analytical approximation can be attributed to saturation of $Ca^{2+}$ binding sites in MC simulations. Since the probability of a $Ca^{2+}$ binding to a site on the target sensor is decreased in the presence of the competing buffer molecules (Fig. 3F), we tested whether the presence of physiological concentrations of EFBs could reduce the difference between the analytical approximation and MC simulations. Indeed, the presence of EFB decreases $P_N(t,r)$ for both CDs (Fig. 5B), as well as the discrepancy between analytical and simulated curves (Fig. 5B, black lines). The error in the time course estimate was still present for shorter CDs, but for the longer CD, the two curves were indistinguishable (Fig. 5B, green lines). The better accuracy in the presence of EFB can be attributed to lower binding probabilities experienced by the target sensor, which is consistent with lower intracellular free [$Ca^{2+}$].

**Figure 6:**
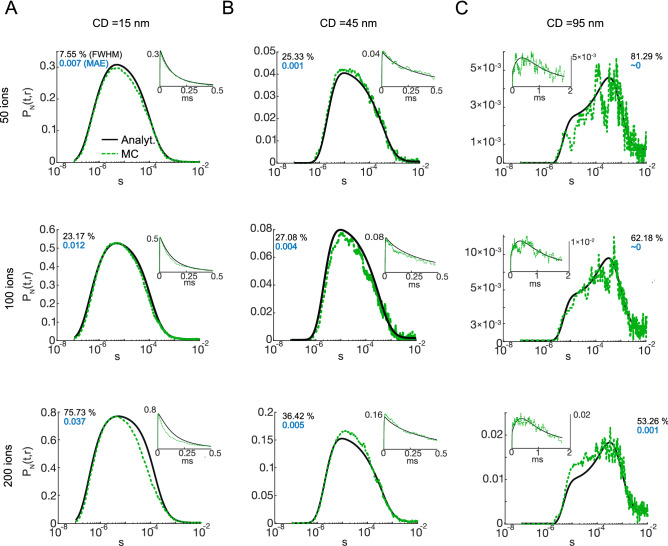
$P_N(t,r)$ for 50 (1st row), 100 (2nd row) and 200 (3rd row) simultaneously released ions for CD of 15 nm (**A**), 45 nm (**B**) and 95 nm (**C**). Main plots represent semi-log scale, while linear scale plots are on insets. Black and green lines show respectively analytical and MC results. The black and blue inset text on each plot represent FWHM error and MAE between analytical and MC results correspondingly. All these computations were realized in the presence of EFB (4 mM).

Because both the presence of a competing $Ca^{2+}$ buffer and increasing the distance to the target could reduce the occupancy probability, we next explored how the number of injected $Ca^{2+}$ ions and unbinding kinetics influenced the discrepancy between MC and analytical $P_N(t,r)$ solutions. The instantaneous $Ca^{2+}$ influx was varied (50, 100, and 200 ions) and the difference between MC and analytical curves was quantified by the Mean Absolute difference (MAE) and full width at half maximum (FWHM) error. We saw that with a decreasing number of released ions, the dissimilarity decreases for all CDs, reflected in the values of MAE and FWHM error (Fig. 6). However, for long CDs we notice a decrease in the FWHM error with an increasing number of injected ions (from 81$\%$ for 50 ions to 53$\%$ for 200 ions) (Fig. 6A,C). This was due to reduced trial variability in MC simulations arising from a higher $P_N(t,r)$. Moreover, altering $P_N(t,r)$ by adjusting $k_{\mathrm{off}}$ (slow ($0.157~{\text {ms}}^{-1}$) and fast ($1570~{\text {ms}}^{-1}$)) was also consistent with the primary source of error being due to high occupancy (see also Figs. 1 and 2 in the Supplementary Information (SI)).

### Analytical solution for computing sensor occupancy in response to $Ca^{2+}$ fluxes generated by stochastic VGCCs

**Figure 7:**
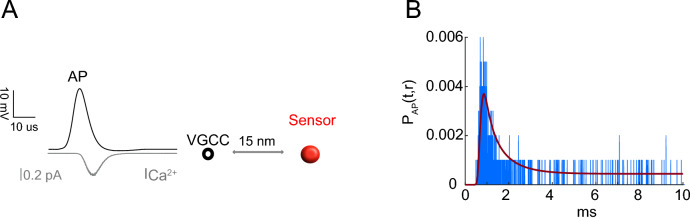
$Ca^{2+}$ entry through a stochastic VGCC. (**A**) Opening of the single VGCC is driven by an AP (black line) triggering $Ca^{2+}$ influx (gray line). Released $Ca^{2+}$ ions diffuse towards a sensor at distance of 15 nm. (**B**) The $P_{AP}(t,r)$ computed using analytical approximation (red) and MC (blue).

Thus far, we considered the instantaneous entry of $Ca^{2+}$ ions. However, it is known that presynaptic $Ca^{2+}$ fluxes arise from a temporally distributed opening of VGCCs during an AP, lasting hundreds of microseconds (Fig. 7A). Moreover, it is also known that accurate estimates of sensor occupancy probability must consider the stochastic nature of VGCC openings. Here we studied the analytical solution for $Ca^{2+}$ fluxes generated from the stochastic opening of VGCCs ($P_{AP}(t,r)$; see “Methods” section). The VGCC model was constrained by experimental estimates of single-channel open probability, single-channel conductance, and duration of the current^20^ (see “Methods” section and Section V of the SI). In each trial of MC simulation, a random realization of VGCC opening and associated $Ca^{2+}$ fluxes were generated as a sequence of entry times of $Ca^{2+}$ ions. Using the analytical solution, we calculated the occupancy probability *P*(*t*, *r*) for each $Ca^{2+}$ entry time, and got then the occupancy probability for at least one ion among all the entered ions. The occupancy probability $P_{AP}(t,r)$ was calculated as the average over 1000 trials of this simulation (see Eq. (28) in “Methods” section).

For a single VGCC located 15 nm from the sensor (Fig. 7A), in the presence of EFB, the calculated $P_{AP}(t,r)$ was similar to that from MC simulations (Fig. 7B, based on 1000 MC trials). The spike-like character of the simulated curve is the consequence of a finite number of trials. The number of bound calcium ions for each trial is zero most of the time, except for a few short periods when it switches to 1. As the average duration of such periods is $1/k_{\mathrm{off}}\approx 0.06$ ms, they look as narrow spikes on Fig. 7B showing the time range between 0 and 10 ms. Moreover, since the binding times are random, the binding periods from different trials typically do not overlap and thus produce multiple individual spikes, except near the maximum of $P_{AP}(t,r)$, where they can superimpose upon the averaging. To eliminate such a discrete character, one would need to increase the number of trials considerably (up to $10^5$ or even $10^6$), which is computationally expensive. In contrast, the theoretical curve, obtained with only 1000 trials, is smooth because the averaging over infinitely many realizations is intrinsically incorporated into the notion of probability.

The peak occupancy is two orders of magnitude smaller than that from the earlier calculation for an instantaneous entry of 200 ions (Fig. 6A), suggesting that the errors due to multiple $Ca^{2+}$ binding are minimal under physiological conditions where the ionic flux occurs over hundreds of microseconds. The extension to multiple channels is trivial using the principle of superposition^13^, provided the total sensor occupancy remains small enough. Thus our analytical method can describe how a simplified SV fusion sensor could be driven by stochastic $Ca^{2+}$ entry during channel openings.

### The occupancy probability for binding at least *n* ions

Seminal experiments at the frog neuromuscular synapse showed a nonlinear relationship between extracellular $[Ca^{2+}]$ and neurotransmitter release, which could be described by a Hill coefficient of  4 (Ref.^32^). More recent evidence suggested that this nonlinearity could be due, in part, to the multi-site occupancy of the sensor protein, synaptotagmin-1, for $Ca^{2+}$^33^. Measurements of single AP-evoked neurotransmitter release are well explained by a 5-state release model^21,34,35^ (see “Methods” section). However, a recent mammalian release model indicated that steep Hill coefficients between intracellular $[Ca^{2+}]$ and SV fusion could also result from the independent binding of calcium ions to multiple sensors, each having a single binding site^7^, suggesting that cooperativity between binding sites is not required to model the nonlinear relationship between intracellular $[Ca^{2+}]$ and neurotransmitter release. To keep our model analytically tractable, we considered the latter sensor model. We derived an analytical solution for the occupancy probability, $P_{N,n}(t,r)$, that at least *n* calcium ions are bound to the sensor at time *t*, given that *N* calcium ions were released simultaneously at time 0 (see Eq. (26) of “Methods” section). If the kinetic rate constants of binding sites were identical and independent, then the $P_{N,n}(t,r)$ would be identical to the occupancy probability of at least *n* binding sites of the sensor at time *t*.

**Figure 8:**
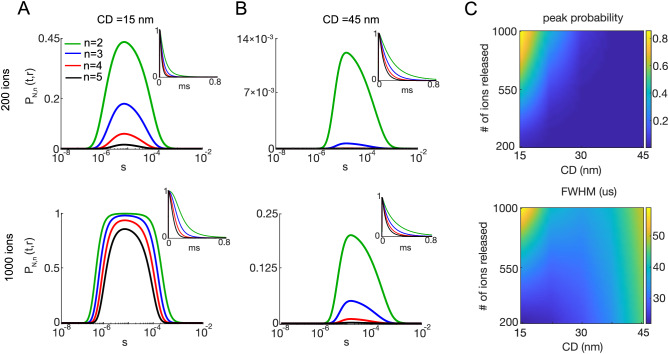
The occupancy probability $P_{N,n}(t,r)$ of at least *n* binding sites on the sensor, given various numbers of ions (*N*) and coupling distances (CD). (**A, B**) $P_{N,n}(t,r)$ for $N = 200$ (1st row) and $N = 1000$ (2nd row) simultaneously released ions for CD of 15 nm (**A**) and 45 nm (**B**), for 2 (green), 3 (blue), 4 (red) and 5 (black) binding sites. (**C**) Peak (top) and FWHM (bottom) of the occupancy probability $P_{N,n}(t,r)$ for $n=5$ independent binding sites, depending on the number of released calcium ions and coupling distance.

Figure 8 summarizes the effect of changing the number *n* of bound calcium ions on the estimated occupancy of the *n*th site $P_{N,n}(t,r)$. As expected, the occupancy probability $P_{N,n}(t,r)$ decreased as *n* increased for short and long coupling distances (Fig. 8A,B). The width of $P_{N,n}(t,r)$ for larger *n* was also narrowed. Taking advantage of the analytical approach, we next examine the amplitude and the width of $P_{N,n}(t,r)$ for various numbers of injected calcium ions and coupling distances. The peak of $P_{N,n}(t,r)$ increased when the number *N* of simultaneously fluxed calcium ions is increased; in turn, this peak decreased when the coupling distance increased (Fig. 8C). The width of the occupancy probability for five binding sites shows a decreased sensitivity to increasing *N* as compared with the single binding site results reported in Fig. 6, where the highest discrepancy between MC and analytical results was observed for large occupancy probabilities, as reflected by the larger difference in the FWHM between MC and analytical curves (Fig. 6A (bottom plot)). We emphasize that obtaining such a contour plot by Monte Carlo simulations would take approximately eight months of computation on 250 CPUs.

Compared with Fig. 6, $P_{N,n}(t,r)$ for $n=5$ remains below the occupancy of 0.5 for up to 500 instantaneously fluxed ions at 15 nm. Thus these analytical calculations show that when considering the multi-site binding $Ca^{2+}$ sensor proteins, it is possible to estimate the occupancy of the fully bound sensor without saturation for an influx mediated by a point flux equivalent to that of two simultaneously open channels (500 ions in total) and a coupling distance of 15 nm. All the simulations taken together, we demonstrate that our first passage-based analytical solution can account for simple multi-site sensors and $Ca^{2+}$ fluxes in the physiological range. However, our approach does not account for nonlinearities arising from cooperative alterations in the binding constants during sequential binding events, which could be a topic for further study in the future.

## Discussion and conclusion

In this work, we introduced an analytical framework for computing the $Ca^{2+}$ occupancy of a target protein sensor for SV fusion following molecular diffusion from a voltage-gated calcium channel source. The main novelty of this approach is its ability to account for binding and unbinding kinetics of the sensor in the presence of competing $Ca^{2+}$ buffers. Moreover, the unbinding kinetics that were ignored in former analytical studies, possibly due to theoretical challenges of its implementation^17^, were shown to be a major factor that shapes the sensor occupancy probability. Our first-passage approach obviates the need to perform computationally intensive MC simulations and preserves the accuracy in predicting biological stochasticity.

We first analyzed the sensor occupancy probability *P*(*t*, *r*) for a single $Ca^{2+}$ and its dependence on various diffusion–reaction parameters. In particular, we observed that the peak of *P*(*t*, *r*) was usually at a few tens of microseconds, a timescale comparable to experimental measurements of SV fusion times, but a thousand times smaller than the mean first-passage time, which gives a measure of the average rate occurrence of a stochastic binding. Calcium ion diffusion and reaction with a sensor is thus a striking biological example of a process in which the mean first-passage time is misleading, whereas the first-passage time distribution cannot be reduced to its mean. For calculating sensor occupancy in response to a flux of *N* independent $Ca^{2+}$, we showed by comparing to MC simulations that $P_N(t,r)$ was accurate, provided the peak occupancy probability was less than 0.5. In particular high $P_N(t,r)$ are achieved with large molecular fluxes and short CDs ($<20$ nm). The peak $P_N(t,r)$ was reduced by including competing $Ca^{2+}$ buffers, thereby increasing the number of $Ca^{2+}$ that could be simulated accurately. While future mathematical studies are needed to account for molecular occlusion that occurs when *P*(*t*, *r*) is high (>0.5), the current analytical solution is accurate for various conditions such as low $Ca^{2+}$ fluxes (corresponding to 0.3 ms single-channel currents of 0.3 pA), long CDs (> 40 nm), and in the presence of the EFB. The solution was also shown to be accurate for the case of $Ca^{2+}$ fluxes via single stochastic VGCC, even at 15 nm. This is likely due to the lower instantaneous flux that occurs for time-distributed influx during an action potential.

Because the $Ca^{2+}$ dependence of SV fusion is known to be nonlinear (Hill coefficient = 4), we then considered the occupancy of several independent binding sites of the sensor for SV fusion^7^, which we implemented using a simple combinatorial calculation. This allowed us to calculate the occupancy probability $P_{N,n}(t,r)$ by at least *n* calcium ions, i.e., the probability that *n* (or more) calcium ions are simultaneously bound to the sensor at time *t*. We showed that the duration of $P_{N,n}(t,r)$ became briefer, meaning that there is a relatively narrow time window during which the SV fusion is possible, as has been observed experimentally^4^.

Several extensions of the current model and solution are possible. First, one can include multiple SV sensors located in different regions of the synaptic bouton. For this purpose, one can partition the synaptic membrane into “zones of influence” around each sensor, as proposed earlier^18,36^; as a calcium ion released within the zone of influence of a given sensor would have more chances to bind to that particular sensor, the binding dynamics within each zone of influence can be studied separately from the others, at least in a first approximation. Second, our analytical solution describes the occupancy probability for a single sensor that can bind a very large (strictly speaking, unlimited) number of $Ca^{2+}$. Accounting for the saturation of the sensor's binding sites is another important perspective that can be explored by adapting recently proposed models for the dynamics of impatient particles^17,19^. In particular, Lawley and Madrid suggested modeling the distribution of the first-passage time to a target by a mono-exponential function, in which case the number of bound particles can be described by a Markov birth-death process, for which the first-passage time statistics are well known^19^. However, the accuracy of such an approximation remains questionable in our setting, especially for re-binding steps when the particle unbinds from the sensor and diffuses in bulk until the next binding. Once the bound $Ca^{2+}$ occlusion problem is correctly implemented, it would then be feasible to investigate the cooperative behavior generated by multiple binding sites.

In conclusion, our analytical solution allows researchers to rapidly explore the vast parameters space of the vesicle release process that includes: the binding/unbinding rates of the sensor and multiple buffers, diffusion coefficients of calcium ions in free and bound states, the sizes of the sensor and the synaptic bouton, the spatial arrangement of multiple VGCCs (or the coupling distance in the case of a single VGCC), the number and the temporal release profile of calcium ions of each VGCC, and the number of binding sites on the sensor. For any physiologically relevant configuration of these parameters, the occupancy probability over a comprehensive range of timescales (from nanosecond to second) can now be calculated almost instantly, as compared to conventional deterministic or particle-based simulations. For instance, the simulation in Fig. 5B took 96 h to simulate the black curve using MC methods and 250 CPUs. In contrast, the analytical method took less than a minute to calculate on a laptop with a 1.7 GHz Intel Core i5. Furthermore, the analytical nature of the solution does not generate integration errors from small time-steps, which is detrimental for long time scales when using conventional numerical techniques. Moreover, the explicit calculation of the occupancy probability for a single calcium ion enables accurate simulation of rare stochastic events.

More generally, our mean-field approach circumvents the limitations of tracking the diffusion of many thousands of molecules that represent the millimolar concentrations found in biology. The time efficiency of our method is vastly superior to the existing MC alternatives. In contrast, the analytical solution gave essentially the same results as MC in a fraction of a minute, without limitations on molecular concentrations. This opens doors to the exploration of complex diffusion–reaction systems that were previously out of reach. In this way, one can discover some specific regions of biological interest in the parameters space. For instance, Fig. 8C (top) showed the dependence of the peak of the occupancy probability on the number of fluxed $Ca^{2+}$ and the coupling distance. In this figure, we showed the range of values of *N* and CD that produced occupancy probabilities high enough to fully occupy the sensor. Plotting a similar figure with conventional simulations would be extremely long, especially due to such combinations of parameters that produce very small occupancy probabilities. At the same time, the derived analytical solution relies on a simplified model of $Ca^{2+}$ binding to the sensor for SV fusion, which neglects some biophysical aspects of this sophisticated process. Even if more advanced models cannot be solved analytically, yet, they can be analyzed by modern simulators. As a consequence, improved performance can be achieved by combining the present analytical approach with conventional numerical simulations.

## Methods

### Theoretical model

(i) **Geometric settings**. A $Ca^{2+}$ ion (or multiple ions) was injected on the membrane on the distance *r* from the center of a synaptic terminal (bouton) with radius R, and allowed to diffuse throughout. At the center, we placed a single $Ca^{2+}$ sensor of hemispherical shape and radius $\rho$, a value analogous to an interaction radius (Fig. 2A). The outer boundary of the bouton (a hemi-spere of radius *R*) is modeled as reflecting, i.e., the flux of $Ca^{2+}$ ions at this boundary is zero. Note that more elaborate partially reflecting boundary could also be considered to account for $Ca^{2+}$ ions escaping far from the synaptic bouton membrane but we strick to the reflecting condition here. In summary, we consider the active zone of the shape (Fig. 2B)
  1 $$\begin{aligned} \Omega _0= \{ \varvec{x}=(x,y,z) \in {\mathbb {R}}^3 :\rho<|\varvec{x}|<R,~ z>0 \} . \end{aligned}$$
  Importantly, we neglect the presence of the synaptic vesicle whose reflecting boundary might hinder the motion of $Ca^{2+}$ ions; in fact, it has been shown by Monte Carlo simulations that in physiological conditions, the synaptic vesicle does not influence the single vesicle release probability^14^.
(ii) ***Ca***^**2+**^ **ions** are modeled as independent point-like diffusing particles that undergo Brownian motion with diffusion coefficient $D_0$ in the region $\Omega _0$ between the boundaries of sensor and active zone; in particular, the charge of $Ca^{2+}$ ions is ignored due to bulk screening of electrostatic interactions. A $Ca^{2+}$ ion source (VGCC) was set at a fixed distance $r-\rho$ from the sensor (we discuss below how to deal with multiple sources).
(iii) **Buffers** are modeled as co-existing continuous homogeneous reactive media that can bind, transport, and release $Ca^{2+}$ ions; their functioning is assumed to be in a linear regime (i.e., low occupancy), i.e. the exchange between the free $Ca^{2+}$ state (denoted by index 0) and the bound state with the *i*-th buffer (denoted by index *i*) occurs through the standard first-order kinetics, with the exchange rates $k_{0i}$ and $k_{i0}$; the $Ca^{2+}$ ion in a bound state diffuses with the diffusion coefficient $D_i$ but cannot bind to the sensor. Under the assumption of a homogeneous reactive medium, the “binding rate” can be expressed as $k_{0i} = k_{{\mathrm{on}},i} \, c_i$, where $k_{{\mathrm{on}},i}$ is the conventional binding constant and $c_i$ is the concentration of the *i*-th buffer.
(iv) **Sensor kinetics**. In a basic setting, we consider a sensor with a single binding site, its kinetics is determined by $k_{\mathrm{on}}$ and $k_{\mathrm{off}}$ binding rate constants. In most cases (unless stated otherwise), we used reaction rate constants identical to the reaction rate constant of the first binding site from the 5 state sensor model^21^ (see also below). When the $Ca^{2+}$ ion reaches the surface of the sensor it can be reflected from it or bind to it, the random choice of either depending on the sensor binding constant $k_{\mathrm{on}}$^37,38^. When bound, the $Ca^{2+}$ ion remains in this state for a random time $\tau$ distributed by an exponential law
  2 $$\begin{aligned} \Phi (t) = {\mathbb {P}}\{\tau >t\} = \exp (-k_{{\mathrm{off}}}\, t), \end{aligned}$$
  $1/k_{\mathrm{off}}$ being the mean waiting time before unbinding reaction. After the unbinding from the sensor, the $Ca^{2+}$ ion resumes its diffusion in the intrasynaptic region $\Omega _0$ until the next binding event.

As any model, our theoretical description is based on assumptions, in which some reactions were simplified and others neglected. For instance, we ignored $Ca^{2+}$ extrusion mechanisms that exist in the synapse to return the $Ca^{2+}$ level to baseline within hundreds of milliseconds^39^. Even though these mechanisms could affect our results for timescales greater than $t \gtrsim 0.1$  s, they are irrelevant in the microsecond/millisecond range, at which the occupancy probability is maximal and thus the SV fusion would most likely occur. We note that these mechanisms could be introduced into our model via reversible reactions with an additional (artificial) buffer. We also neglected the baseline $Ca^{2+}$ level that plays important roles, e.g. for spontaneous SV release^40^. This residual level of resting $Ca^{2+}$ ions can be included into our diffusion–reaction equations as an appropriate initial condition (e.g., with a uniform concentration) or via a source term on the outer boundary of the synaptic bouton. The effect of this baseline level onto the occupancy probability and the consequent spontaneous SV release can be further investigated. Perhaps, the most significant simplification is the assumption of unlimited binding capacity of the sensor and the consequent consideration of multiple $Ca^{2+}$ binding events as independent (see below). This simplification allows for the detailed examination of spatio-temporal relationship between nanoscale $[Ca^{2+}]$ gradients and their ability to drive nonlinear SV fusion reactions - both critical features of synaptic transmission.

### Sensor occupancy probability

We are interested in computing the so-called occupancy probability $P(t,\varvec{x})$ that a particle (here, a $Ca^{2+}$ ion), started from a fixed point $\varvec{x}$ at time 0, is at the bound state on the sensor at a later time *t*. Due to the sensor kinetics, the particle can undergo numerous binding/unbinding events up to time *t*. To account for these events, we introduce an auxiliary probability density $\psi _n(t,\varvec{x})$ of the *n*-th binding at time *t*. These densities can be obtained via recurrent functional relations. In fact, the independence between the time spent in the bound state on the sensor, and the time of a bulk excursion after unbinding, implies

3 $$\begin{aligned} \psi _n(t,\varvec{x}) = \int \limits _0^t dt_1 \int \limits _{t_1}^t dt_2 \, \psi _{n-1}(t_1,\varvec{x}) \, \phi (t_2-t_1) \, \psi (t-t_2) , \end{aligned}$$

where $\phi (t) = k_{\mathrm{off}}\, \exp (-k_{\mathrm{off}}\, t)$ is the probability density of the exponential waiting time in the sensor-bound state, and $\psi (t)$ is the probability density of re-binding at time *t* after the release at time 0. This is a standard renewal relation, which states that, after the $(n-1)$-th binding of the particle at some time $t_1$ (with the density $\psi _{n-1}(t_1,\varvec{x})$), the particle remains bound during time $t_2-t_1$ and unbinds at time $t_2$ (with the density $\phi (t_2-t_1)$), diffuses in the bulk during time $t-t_2$ and re-binds at time *t* (with the density $\psi (t-t_2)$). Since the intermediate binding/unbinding events may occur at any times between 0 and *t*, one has to integrate over $t_1$ and $t_2$. The integral relation (3) is reduced to a product in the Laplace space, i.e.,

4 $$\begin{aligned} {\tilde{\psi }}_n(p,\varvec{x}) = {\tilde{\psi }}_{n-1}(p,\varvec{x}) \, {\tilde{\phi }}(p) \, {\tilde{\psi }}(p) = {\tilde{\psi }}_1(p,\varvec{x}) \bigl [{\tilde{\phi }}(p) \, {\tilde{\psi }}(p)\bigr ]^{n-1}, \end{aligned}$$

where tilde denotes Laplace-transformed quantities, e.g.,

5 $$\begin{aligned} {\tilde{\psi }}_n(p,\varvec{x}) = \int \limits _0^\infty dt \, e^{-pt} \, \psi _n(t,\varvec{x}). \end{aligned}$$

The probability of a particle to be in the bound state at time *t* can be expressed as follows

6 $$\begin{aligned} P(t,\varvec{x}) = \sum \limits _{n=1}^\infty \int \limits _0^t dt' \, \psi _n(t',\varvec{x}) \, \Phi (t-t') . \end{aligned}$$

In this infinite sum, the *n*-th term is the probability that after the *n*-th binding at time $t'$ (with the density $\psi _n(t',\varvec{x})$), the particle remains at the bound state for time $t-t'$ (with the probability $\Phi (t-t')$ given by Eq. (2)). This relation simply reflects the fact that the particle, which is at the bound state at time *t*, has experienced either 1, or 2, $\ldots$ or *n*, or $\ldots$ binding events. In the Laplace space, we get

7 $$\begin{aligned} {\tilde{P}}(p,\varvec{x}) = \sum \limits _{n=1}^\infty {\tilde{\psi }}_n(p,\varvec{x})\, {\tilde{\Phi }}(p) = {\tilde{\psi }}_1(p,\varvec{x}) \biggl (1 - {\tilde{\phi }}(p) \, {\tilde{\psi }}(p)\biggr )^{-1} {\tilde{\Phi }}(p) . \end{aligned}$$

The three factors in the product have a clear interpretation: the first arrival and binding onto the sensor, multiple re-binding events on the sensor, and waiting after the last re-binding. For exponential waiting times, one has ${\tilde{\Phi }}(p) = 1/(p+k_{\mathrm{off}})$ and ${\tilde{\phi }}(p) = k_{\mathrm{off}}/(p+k_{\mathrm{off}})$, and thus we finally get

8 $$\begin{aligned} {\tilde{P}}(p,\varvec{x}) = {\tilde{\psi }}_1(p,\varvec{x}) \, \biggl (p + k_{\mathrm{off}}(1-{\tilde{\psi }}(p))\biggr )^{-1} . \end{aligned}$$

The inverse Laplace transform of Eq. (8) allows one to return to the time domain to get $P(t,\varvec{x})$. In this way, the probability $P(t,\varvec{x})$ is reduced to the analysis of the “elementary” diffusion step – the binding to the sensor – that determines both ${\tilde{\psi }}_1(p,\varvec{x})$ and ${\tilde{\psi }}(p)$. We emphasize that Eq. (8), written in terms of survival probabilities, is well known for describing reversible kinetics in chemical physics, see Ref.^41,42^ and references therein.

### Distribution of the first-binding time

We start by noting that the symmetry of the considered domain $\Omega _0$ allows one to effectively remove the synaptic bouton membrane at $z = 0$ and thus replace $\Omega _0$ by a simpler spherical layer

9 $$\begin{aligned} \Omega = \{ \varvec{x}= (x,y,z) \in {{\mathbb {R}}}^3:~ \rho< |\varvec{x}| < R \}. \end{aligned}$$

In other words, the distribution of first-passage times computed in $\Omega _0$ is identical to that computed in $\Omega$. The advantage of the latter domain is that it is rotation invariant so that the problem can be reduced to one-dimensional radial part, as discussed below. When there is no buffer, the computation of the first-passage time to a target is rather standard^43–45^ but technically involved in the case of a spherical layer^46^. Accounting for buffers presents one of the major challenges and originalities of this work. Note that our approach generalizes some earlier results for two-channel diffusion^47^.

We investigate the model with *M* distinct buffers by using an $(M+1)$-state switching diffusion model: the $Ca^{2+}$ ion can be either in a free state (0) or in a buffer-bound state (*i*), with $i=1,\ldots ,M$. Given that the buffers are modeled as continuous and homogeneous media, a transition from the state *i* to the state *j* happens spontaneously, with a given rate $k_{ij}$ (see Section I of the SI for a formal definition of the model). A general scheme for studying first-passage times for switching diffusions was recently developed in Ref.^24^. We introduce $(M+1)$ survival probabilities $S_i(t,\varvec{x})$ for a $Ca^{2+}$ ion started at $\varvec{x}$ in the state *i* to be unbound from the sensor until time *t*. These probabilities satisfy $(M+1)$ coupled backward Fokker-Planck (or Kolmogorov) equations^23^:

10 $$\begin{aligned} \partial _t S_i = D_i \Delta S_i + \sum \limits _{j=0}^M k_{ij} (S_j - S_i) \quad (i = 0,1,\ldots ,M), \end{aligned}$$

subject to the initial condition: $S_i(0,\varvec{x}) = 1$. Here $D_i$ is the diffusion coefficient of the $Ca^{2+}$ ion in the state *i*, $\Delta$ is the Laplace operator, and we set $k_{ii} = 0$ to simplify notations. We recall that there is no direct $Ca^{2+}$ ion exchange between bound states:

11 $$\begin{aligned} k_{ij} = 0 \qquad (1\le i,j\le M). \end{aligned}$$

In other words, any exchange between the states *i* and *j* occurs through the free state 0. The last term in Eq. (10) describes transitions between states *i* and *j*.

Equations (10) should be completed by boundary conditions at the inner sphere at $|\varvec{x}| = \rho$ (the sensor) and the outer sphere at $|\varvec{x}| = R$ (the frontier of the active zone). The outer reflecting boundary simply confines the $Ca^{2+}$ ions within the active zone, i.e., it ensures that there is no flux of $Ca^{2+}$ ions across this boundary:

12 $$\begin{aligned} - D_i \partial _n S_i(t,\varvec{x}) = 0 \quad (|\varvec{x}|=R, ~ i=0,1,\ldots ,M), \end{aligned}$$

where $\partial _n$ is the normal derivative directed outwards the domain. Since the $Ca^{2+}$ ions in bound states cannot bind to the sensor, the same Neumann boundary condition is imposed at the inner sphere:

13 $$\begin{aligned} - D_i \partial _n S_i(t,\varvec{x}) = 0 \quad (|\varvec{x}|=\rho , ~ i=1,\ldots ,M). \end{aligned}$$

Finally, the calcuim ions in the free state can bind to the sensor that implies the Robin boundary condition

14 $$\begin{aligned} - D_0 \partial _n S_0(t,\varvec{x}) = \frac{k_{\mathrm{on}}S_0(t,\varvec{x})}{N_A (4\pi \rho ^2)} \quad (|\varvec{x}|=\rho ) . \end{aligned}$$

It is obtained by equating the net diffusive flux at the sensor (left-hand side) to the reactive flux (right-hand side) controlled by the reaction constant $k_{\mathrm{on}}$, where $N_A$ is the Avogadro number, and $4\pi \rho ^2$ is the surface area of the sensor^48,49^. We emphasize that the presence of buffers has two effects: change in the diffusion coefficient and impossibility of a buffer-bound $Ca^{2+}$ ion to bind to the sensor. Since the calcuim ions are released in the free state, we are interested exclusively in $S_0(t,\varvec{x})$. However, finding this probability requires solving the coupled system of equations for all $S_i$. Note that $1 - S_0(t,\varvec{x})$ describes the fraction of $Ca^{2+}$ ions that have been bound to the sensor up to time *t*. This is the cumulative probability distribution for the first-binding time. In particular, its probability density reads

15 $$\begin{aligned} \psi _1(t,\varvec{x}) = \partial _t \bigl (1 - S_0(t,\varvec{x})\bigr ) = - \partial _t S_0(t,\varvec{x}) . \end{aligned}$$

Due to the rotational invariance of the problem, the probabilities $S_i(t,\varvec{x})$ and the probability density $\psi _1(t,\varvec{x})$, written in spherical coordinates, depend only on the radial coordinate $r = |\varvec{x}|$. From now on, we replace $\varvec{x}$ by *r*.

The solution of the system (10) of coupled partial differential equations is detailed in Section II of the SI. In a nutshell, the Laplace transform reduces these equations to a system of ordinary differential equations with respect to the radial coordinate *r* that is then solved by standard methods. Once the solution is found, one gets from Eq. (15)

16 $$\begin{aligned} {\tilde{\psi }}_1(p,r) = 1 - p {\tilde{S}}_0(p,r) . \end{aligned}$$

In addition, as a bulk excursion after the unbinding event starts at the sensor surface, $r = \rho$, one has

17 $$\begin{aligned} {\tilde{\psi }}(p) = {\tilde{\psi }}_1(p, \rho ) . \end{aligned}$$

As a consequence, the knowledge of ${\tilde{S}}_0(p,r)$ yields both dynamical characteristics, ${\tilde{\psi }}_1(p,r)$ and ${\tilde{\psi }}(p)$, that determine the Laplace-transformed probability ${\tilde{P}}(p,r)$ according to Eq. (8).

The last step for getting *P*(*t*, *r*) in time domain requires the inverse Laplace transform of ${\tilde{P}}(p,r)$. This is performed by determining the poles of this function and applying the residue theorem. When the poles are simple, the occupancy probability admits the following exact representation:

18 $$\begin{aligned} P(t,r) = P_\infty + \sum \limits _{n=1}^\infty \exp \bigl (- \alpha _n^2 D_0 t/\rho ^2 \bigr ) \sum \limits _{j=0}^M b_n^{(j)} \, u(\alpha _n^{(j)},r) , \end{aligned}$$

where

19 $$\begin{aligned} u(\alpha , r) = \frac{\rho \, \sin \bigl (\alpha \frac{R-r}{\rho }\bigr ) - R\alpha \cos \bigl (\alpha \frac{R-r}{\rho }\bigr )}{r} \,, \end{aligned}$$

and

20 $$\begin{aligned} P_\infty = \biggl (1 + k_{\mathrm{off}}\frac{4\pi (R^3 - \rho ^3)N_A}{3k_{\mathrm{on}}} \biggl (1 + \sum \limits _{j=1}^M \frac{k_{0j}}{k_{j0}}\biggr ) \biggr )^{-1} \end{aligned}$$

is the steady-state limit. The coefficients $b_n^{(j)}$ and $\alpha _n^{(j)}$ are determined by exact but complicated formulas provided in the SI, whereas $\alpha _n$ are found as strictly positive solutions of some trigonometric equation (provided in the SI). For instance, in the simplest case of no buffer ($M = 0$), we obtained in Section III of the SI:

21 $$\begin{aligned} \alpha _n^{(0)} = \alpha _n, \qquad b_n^{(0)} = \frac{2 \mu }{\sin (\alpha _n \beta ) \bigl (\alpha _n^2 w_1 + w_2 \bigr ) + \alpha _n \cos (\alpha _n\beta ) \bigl (\alpha _n^2 w_3 + w_4 \bigr )} , \end{aligned}$$

where

22a $$\begin{aligned} w_1&= 4(1+\beta ) + \beta (\beta +\mu (1+\beta )) , \end{aligned}$$

22b $$\begin{aligned} w_2&= 2(1+\mu -\lambda (1+\beta )) - \lambda \beta ^2 , \end{aligned}$$

22c $$\begin{aligned} w_3&= \beta (1+\beta ) , \end{aligned}$$

22d $$\begin{aligned} w_4&= \beta (1 + \mu - \lambda (1+\beta )) - 3(\beta + \mu (1+\beta )) , \end{aligned}$$

with dimensionless parameters

23 $$\begin{aligned} \beta = (R - \rho )/\rho , \qquad \lambda = k_{\mathrm{off}}\rho ^2/D_0 , \qquad \mu = k_{\mathrm{on}}/(4\pi \rho D_0 N_A), \end{aligned}$$

and $\alpha _n$ are strictly positive solutions of the trigonometric equation

24 $$\begin{aligned} \sin (\alpha _n \beta ) = \frac{\bigl [\alpha _n^2(\beta +\mu (1+\beta )) - \lambda \beta \bigr ] \alpha _n \cos (\alpha _n \beta )}{\alpha _n^4(1+\beta ) + \alpha _n^2(1+\mu - \lambda (1+\beta )) - \lambda } \qquad (n=1,2,\ldots ). \end{aligned}$$

For a single buffer ($M = 1$), we also derived explicit formulas but they are much more cumbersome (see Section IV of the SI). Even though analytical calculations become prohibitively complicated for $M > 1$, numerical computations based on our analytical solution remain fast and accurate.

The exact solution (18) is the main analytical result of the paper. Although this solution may look cumbersome and involves some numerical steps (truncation of the infinite series, numerical computation of the coefficients, etc), its explicit form allows for both analytical and numerical investigation of the occupancy probability *P*(*t*, *r*).

### Instant calcium influx

If *N* independent ions are released simultaneously from the same fixed position *r*, then the single binding site occupancy probability can be computed as a probability of at least one out of *N* ions being bound to the sensor, according to the formula:

25 $$\begin{aligned} P_N(t,r)= 1-(1-P(t,r))^N, \end{aligned}$$

where *P*(*t*, *r*) is the occupancy probability of single binding site by a single ion. The above formula relies on the assumption that the sensor has an unlimited binding capacity. In other words, the sensor can simultaneously bind $1,2,3,\ldots ,N$ calcium ions, and its binding rate $k_{\mathrm{on}}$ does not depend on the number of already bound ions. This assumption was crucial to be able to consider the reaction kinetics of *N* calcium ions independently from each other and thus to reduce the original very complicated “*N*-body problem” to a simple relation (25) involving only the occupancy probability *P*(*t*, *r*) for a single ion. In statistical physics, this would correspond to a mean-field approximation.

Similarly, one can compute the occupancy probability $P_{N,n}(t,r)$ for at least *n* calcium ions bound simultaneously to the sensor at time *t* as

26 $$\begin{aligned} P_{N,n}(t,r) = 1 - \sum _{k=0}^{n-1} \left( {\begin{array}{c}N\\ k\end{array}}\right) \, [P(t,r)]^k \, (1-P(t,r))^{N-k} \,, \end{aligned}$$

where $$\left( {\begin{array}{c}N\\ k\end{array}}\right)$$ is the binomial coefficient. For $n = 1$, this formula is reduced to Eq. (25) for $P_N(t,r)$. If there was no unbinding kinetics from the sensor (i.e., if $k_{\mathrm{off}}= 0$), $1-P_{N,n}(t,r)$ could be interpreted as the “survival” probability that there were no *n* bound calcium ions up to time *t*. This survival probability would then determine the probability density of the first moment ${{\mathbb {T}}}_n$ when *n* calcium ions are simultaneously bound to the sensor. As calcium ions could not unbind, this is precisely the first moment $\hat{{{\mathbb {T}}}}_n$ of binding of the *n*-th calcium ion: $\hat{{{\mathbb {T}}}}_n = {\mathbb{ T}}_n$. However, the possibility of unbinding (i.e., $k_{\mathrm{off}}> 0$) transforms this equality into inequality, $\hat{{{\mathbb {T}}}}_n < {{\mathbb {T}}}_n$, and makes the computation of ${{\mathbb {T}}}_n$ a challenging open problem. This mathematical difficulty was probably one of the reasons why the unbinding kinetics was ignored in former theoretical works on this topic. Here, we made an important step toward a more realistic analytical model by incorporating the unbinding kinetics into the occupancy probability $P_{N,n}(t,r)$ which can be seen as a proxy for the likelihood of the SV fusion at time *t*.

In the MC simulations $P_N(t,r)$ was computed as the probability of finding a single ion bound to the sensor, given that *N* ions were released at the same time from the same position.

### Calcium influx through single VGCC

Single VGCC was modeled as a three-step Hodgkin-Huxley process^50^ (see also Section V the SI), the model and parameters are taken Ref.^20^. This model reproduces the single channel characteristics that were measured previously: opening probability 0.3, maximum of the single channel current 0.3 pA, full width half maximum of the single channel current 250 $\upmu$s.

Simulation of $Ca^{2+}$ influx through the channel was done using MC tool, with 1000 trials. For the *j*-th trial, we stored the random times $t_{1}^{(j)}, \ldots , t_{N}^{(j)}$ when *N* ions “entered” the system. Given the instances of ion appearance we calculated the probability of sensor occupancy for this trial using Poisson binomial distribution at each point:

27 $$\begin{aligned} P_{trial}^{(j)}(t,r) = 1 - \prod \limits _{i=1}^N (1-P(t+t_i^{(j)},r)). \end{aligned}$$

Then these probabilities were averaged among the trials:

28 $$\begin{aligned} P_{AP}(t,r) = \frac{1}{1000} \sum _{j=1}^{1000} P_{trial}^{(j)}(t,r) . \end{aligned}$$

More generally, if the ions entered from different VGCC channels, one could use $P(t+t_i^{(j)},r_i^{(j)})$ with the appropriate location $r_i^{(j)}$ of the source of the *i*-th ion in the *j*-th trial. In this way, one can easily implement sophisticated spatio-temporal characteristics of the $Ca^{2+}$ ions release.

The analytical solution from Eq. (28) can be compared to the direct estimate of this probability from Monte Carlo simulations. For each simulation trial, we computed the moments of binding and unbinding of calcium ions that determine the number of bound calcium ions at time *t*, $N^{(j)}(t,r)$, at the trial *j*. The average of these functions over all trials is the direct estimate of the probability:

29 $$\begin{aligned} P_{AP}^{MC}(t,r) = \frac{1}{1000} \sum _{j=1}^{1000} N^{(j)}(t,r) . \end{aligned}$$

The comparison between $P_{AP}(t,r)$ and $P_{AP}^{MC}(t,r)$ is shown on Fig. 7B.

### Stochastic simulations

For verification of analytical results we use particle-based stochastic numerical simulations (MCell software^51^). In MCell diffusion of individual molecules is modeled using Brownian dynamics, while chemical reactions occur due to the collision of molecules and follow Poisson distribution. All the parameters for simulations are identical to the parameters of analytical solution. The presynaptic domain of radius 300 nm and sensors are modeled as spheres, intersected by a reflecting plane in the origin (Fig. 2B). The sensor is located in the origin of the volume and has a radius of 5 nm. Depending on the context of the simulation, the $Ca^{2+}$ input, number of calcium channels and the distance between the sensor and calcium channels were manipulated; for instance, for the computation of the occupancy probability by a single ion, it was released at time 0 from a single source. Each time the particle hits the sensor was recorded. The first-passage time distribution was computed based on the recorded times.

To compute occupancy probabilities we stored the time instances of the reaction between $Ca^{2+}$ ion and the sensor, then the number of binding events at each time instance was divided by the total number of trials. The interaction range between two particles was set to 5 nm, the time step was chosen to be 5 ns.

### Deterministic simulations

The release rates can be simulated using a 5-state model of $Ca^{2+}$ triggered vesicle fusion^21^:

30 $$\begin{aligned} V_0 \mathrel {\mathop {\rightleftarrows }^{5 k_{\mathrm{on}}}_{k_{\mathrm{off}}b^0}} V_1 \mathrel {\mathop {\rightleftarrows }^{4 k_{\mathrm{on}}}_{2 k_{\mathrm{off}}b^1}} V_2 \mathrel {\mathop {\rightleftarrows }^{3 k_{\mathrm{on}}}_{3 k_{\mathrm{off}}b^2}} V_3 \mathrel {\mathop {\rightleftarrows }^{2 k_{\mathrm{on}}}_{4 k_{\mathrm{off}}b^3}} V_4 \mathrel {\mathop {\rightleftarrows }^{k_{\mathrm{on}}}_{5 k_{\mathrm{off}}b^4}} V_5 \mathrel {\mathop {\rightarrow }^{\gamma }} F , \end{aligned}$$

where $V_i$ denote the binding states of the sensor (i.e., the sensor with *i* calcium ions bound, and $V_0$ meaning the unbound state), and *F* is the fused state of the vesicle. The conventional values of the parameters are Ref.^21^: $k_{\mathrm{on}}= 127~{\text {mM}}^{-1}\, {\text {ms}}^{-1}$, $k_{\mathrm{off}}=15.7~{\text {ms}}^{-1}$, $b=0.25$, $\gamma =6~{\text {ms}}^{-1}$. We used these values for plotting Fig. 1. For this purpose, the system of ordinary differential equations describing this model was integrated using forward Euler scheme in a custom Matlab routine. The input $Ca^{2+}$ transients are results of the spatial deterministic simulations for the channel-vesicle arrangement as in Fig. 1, provided by Yukihiro Nakamura. The sensor binding sites were assumed not to alter the free calcium due to their small number.

For MC simulations of single binding site occupancies, which were used to compare with analytical solutions, we used five times $k_{\mathrm{on}}$ ($127~{\text {mM}}^{-1}\, {\text {ms}}^{-1}$; see Table 1).

## Supplementary Information

Supplementary Informations.
