# Supplementary material for: A first-passage approach to diffusion-influenced reversible binding and its insights into nanoscale signaling at the presynapse
Source: Sci Rep. 2021 Mar 8;11:5377. doi: 10.1038/s41598-021-84340-4 (PMC7940439; doi:10.1038/s41598-021-84340-4)
Supplement: Supplementary file 1 — Supplementary Informations. [file 41598_2021_84340_MOESM1_ESM.pdf]

Supplementary Information to the manuscript "A first-passage approach to diffusion-influenced reversible binding and its insights into nanoscale signaling at the presynapse"

Maria Reva[1], David A. DiGregorio[1*], and Denis S. Grebenkov[2*]

[1]Institut Pasteur, Unit of Synapse and Circuit Dynamics, CNRS UMR 3571, Paris, France
[2]Laboratoire de Physique de la Matière Condensée (UMR 7643) , CNRS – Ecole Polytechnique, IP Paris, 91128 Palaiseau, France
[*]senior authors
[**]correspondence: denis.grebenkov@polytechnique.edu and david.digregorio@pasteur.fr

ABSTRACT

In this Supplementary Information, we provide mathematical and technical details of our derivation of the analytic solution for the occupancy probability. We also discuss some additional insights onto this problem.

1 Formal definition of the switching diffusion model

We reproduce here a formal definition of the $(M+1)$-state switching diffusion model following Ref.[1]. We consider a two-component process $(\boldsymbol{X}_t, \nu_t)$, in which $\boldsymbol{X}_t$ is the diffusion process in $\mathbb{R}^3$, and ν_t is the pure jump process with the states at $\{0,1,\ldots,M\}$. When there is no boundary, the process is defined by a standard stochastic equation

$$d\boldsymbol{X}_t = \sqrt{2D_{\nu_t}}\, I\, d\boldsymbol{W}_t, \qquad (\boldsymbol{X}_0, \nu_0) = (\boldsymbol{x}, i), \tag{1}$$

where $\boldsymbol{W}_t$ is the standard Wiener process in $\mathbb{R}^3$, I is the identity matrix, and D_i is the diffusion coefficient at the state i. The jump process is defined for any $i \neq j$ by

$$\mathbb{P}\{\nu_{t+dt} = j \mid \nu_t = i, \boldsymbol{X}_s, \nu_s, s \leq t\} = k_{ij}dt + o(dt), \tag{2}$$

where k_{ij} is the rate of transition from the state i to the state j. The propagator $p(\boldsymbol{x},i,t|\boldsymbol{x}_0,i_0,0)$ is the probability density for the process to be in (the vicinity of) the point $\boldsymbol{x}$ in the state i at time t when stated from the point $\boldsymbol{x}_0$ in the state i_0. The propagator satisfies $(M+1)$ coupled forward Fokker-Planck equations

$$\partial_t p(\boldsymbol{x},i,t|\boldsymbol{x}_0,i_0,0) = D_i \Delta p(\boldsymbol{x},i,t|\boldsymbol{x}_0,i_0,0) + \sum_{j=0}^{M} \big[k_{ji} p(\boldsymbol{x},j,t|\boldsymbol{x}_0,i_0,0) - k_{ij} p(\boldsymbol{x},i,t|\boldsymbol{x}_0,i_0,0)\big], \tag{3}$$

subject to the initial condition $p(\boldsymbol{x},i,0|\boldsymbol{x}_0,i_0,0) = \delta_{i,i_0}\,\delta(\boldsymbol{x}-\boldsymbol{x}_0)$. Some properties of the propagator were discussed in[2–4] (see also the references therein).

In turn, for a given smooth function f, the expectation of a functional $f(\boldsymbol{X}_t, \nu_t)$ given that the process has started at $\boldsymbol{x}$ and i,

$$u(\boldsymbol{x},i,t) = \mathbb{E}\{f(\boldsymbol{X}_t,\nu_t) \mid X_0 = \boldsymbol{x}, \nu_0 = i\}, \tag{4}$$

satisfies the $(M+1)$ coupled backward Fokker-Planck (or Kolmogorov) equations for each i,

$$\partial_t u(\boldsymbol{x},i,t) = D_i \Delta u(\boldsymbol{x},i,t) + \sum_{j=0}^{M} k_{ij}\big(u(\boldsymbol{x},j,t) - u(\boldsymbol{x},i,t)\big), \tag{5}$$

subject to the initial condition $u(\boldsymbol{x},i,0) = f(\boldsymbol{x},i)$ (strictly speaking, this is a terminal condition but as the rates k_{ij} do not depend on time, one can recast it as the initial condition).

In the presence of a (partially) reflecting boundary, the diffusion component of the process is modified in a standard way (via the Skorokhod equation)[5–8], whereas the forward and backward Fokker-Planck equations need to be completed by the associated boundary conditions, see[1–3]. Setting $f = 1$, one can interpret $u(\boldsymbol{x},i,t)$ as the probability for a particle started at $\boldsymbol{x}$ in the state i to survive up to time t.

2 General analytical solution

In this section, we present the derivation of the analytical solution for a general case with M buffers. Two particular cases (without buffer and with one buffer) will be detailed in Sections 3 and 4.

2.1 Survival probabilities

We aim to find the survival probabilities $S_i(t,\boldsymbol{x})$ satisfying Eqs. (5) with $f = 1$ inside the domain

$$\Omega = \{\boldsymbol{x} \in \mathbb{R}^3 \; : \; \rho < |\boldsymbol{x}| < R\} \tag{6}$$

between two concentric spheres of radii ρ and R. The rotation symmetry of this domain implies that $S_i(t,\boldsymbol{x})$ depend only on the radial coordinate $r = |\boldsymbol{x}|$ so that we can drop the dependence on angular coordinates and write $S_i(t,r)$. Equations (5) are subject to the initial condition

$$S_i(t=0,r) = 1, \tag{7}$$

and have to be completed by boundary conditions (see the main text)

$$\rho \big(\partial_r S_i(t,r)\big)_{r=\rho} = \mu_i S_0(t,\rho), \tag{8a}$$

$$\big(\partial_r S_i(t,r)\big)_{r=R} = 0, \tag{8b}$$

at the inner and outer spheres, respectively, where

$$\mu_0 = \mu = \frac{k_{\rm on}}{4\pi\rho D_0 N_A}, \qquad \mu_i = 0 \quad (i = 1,\ldots,M) \tag{9}$$

are dimensionless reactivities, with N_A being the Avogadro number, and $k_{\rm on}$ the on-rate binding constant.

Introducing the Laplace-transformed survival probabilities (denoted by tilde),

$$\tilde{S}_i(p,r) = \int\limits_0^\infty dt\, e^{-pt}\, S_i(t,r), \tag{10}$$

one can rewrite the above equations as

$$(p + k_i - D_i\Delta)\tilde{S}_i - \sum_{j=0}^{M} k_{ij}\tilde{S}_j = 1 \quad (\rho < r < R), \tag{11a}$$

$$\partial_r \tilde{S}_i = 0 \quad (r = R), \tag{11b}$$

$$\mu_i \tilde{S}_i - \rho\, \partial_r \tilde{S}_i = 0 \quad (r = \rho), \tag{11c}$$

where $\Delta = \partial_r^2 + (2/r)\partial_r$ is the radial part of the Laplace operator, and

$$k_i = \sum_{j=0}^{M} k_{ij}. \tag{12}$$

As the rate k_{ii} is undefined, we set $k_{ii} = 0$ for convenience of notations.

We search the Laplace-transformed probabilities in the form

$$\tilde{S}_i(p,r) = a_i + \sum_{j=0}^{M} b_{ij}\, v(\delta_j, r), \tag{13}$$

where a_i and b_{ij} are unknown coefficients, and

$$v(\delta,r) = \frac{\rho}{r}\biggl(\sinh(\delta(R-r)/\rho) - (1+\beta)\delta\cosh(\delta(R-r)/\rho)\biggr), \tag{14}$$

with

$$\beta = (R-\rho)/\rho, \tag{15}$$

and δ_j are unknown factors. In fact, the function $v(\delta,r)$ is a linear combination of two independent solutions $e^{\delta r}/r$ and $e^{-\delta r}/r$ of the equation $\Delta u - \delta^2 u = 0$, and the chosen form (14) ensures the Neumann boundary condition at the outer sphere for any δ:

$$\big(\partial_r v(\delta,r)\big)_{r=R} = 0. \tag{16}$$

Substituting Eq. (13) into Eq. (11), we get for $i = 0,\ldots,M$

$$(p+k_i)\left(a_i + \sum_{j=0}^{M} b_{ij}\, v(\delta_j,r)\right) - \frac{D_i}{\rho^2}\sum_{j=0}^{M} b_{ij}\delta_j^2\, v(\delta_j,r) - \sum_{\ell=0}^{M} k_{i\ell}\left(a_\ell + \sum_{j=0}^{M} b_{\ell j}\, v(\delta_j,r)\right) = 1 \tag{17}$$

Each of these $M+1$ functional relations must be satisfied for any $r\in(\rho,R)$ that implies $M+2$ relations on coefficients for each $i = 0,\ldots,M$:

$$(p+k_i)a_i - \sum_{\ell=0}^{M} k_{i\ell}a_\ell = 1 \tag{18}$$

and

$$\big(p+k_i-(D_i/\rho^2)\delta_j^2\big)b_{ij} - \sum_{\ell=0}^{M} k_{i\ell}b_{\ell j} = 0. \tag{19}$$

The first set (18) of $M+1$ linear equations on a_i is uncoupled from the rest and can be solved separately. Inverting the underlying matrix,

$$W = \begin{pmatrix} \gamma_0 & -k_{01} & -k_{02} & \cdots & -k_{0M} \\ -k_{10} & \gamma_1 & 0 & \cdots & 0 \\ -k_{20} & 0 & \gamma_2 & \cdots & 0 \\ \cdots & \cdots & \cdots & \cdots & \cdots \\ -k_{M0} & 0 & 0 & \cdots & \gamma_M \end{pmatrix} \tag{20}$$

(with $\gamma_i = p+k_i$) and applying to the vector $(1,1,\ldots,1)^\dagger$, one gets

$$a_0 = \left(1+\sum_{i=1}^{M}\frac{k_{0i}}{p+k_{i0}}\right)\left(p+k_0-\sum_{i=1}^{M}\frac{k_{0i}k_{i0}}{p+k_{i0}}\right)^{-1} = \frac{1}{p}. \tag{21}$$

The other a_i can also be found but their contribution will be canceled by $\mu_i = 0$ for $i>0$.

Next, we can treat Eqs. (19) as a set of linear equations on b_{ij}, in which δ_j are some parameters. One can note that, for each j, there are $M+1$ equations whose form does not depend on j. In other words, we can decouple these equations into $M+1$ blocks, each having $M+1$ equations. Let us write δ instead of δ_j for one block. The equations in each block are homogeneous, so that there is either none, or infinitely many solutions. For the existence of solutions, the determinant of the underlying matrix in front of coefficients b_{ij} should be zero. This matrix has precisely the same form as W in Eq. (20), but with $\gamma_i = p+k_i-(D_i/\rho^2)\delta^2$. The determinant of this matrix as a function of $z=\delta^2$ is the polynomial of degree $(M+1)$

$$H(z) = \gamma_1\cdots\gamma_M\left(\gamma_0 - \sum_{i=1}^{M}\frac{k_{0i}k_{i0}}{\gamma_i}\right). \tag{22}$$

The $M+1$ zeros of this polynomial, z_i, determine the unknown δ_i: $\delta_i = \sqrt{z_i}$ (here one can use either of two values $\pm\sqrt{z_i}$, the final results remaining unchanged).

For each j, the set (19) of equations on b_{ij} has infinitely many solutions. One can express b_{ij} (for $i = 1,\ldots,M$) in terms of b_{0j} as

$$b_{ij} = \frac{k_{i0}}{p+k_i-(D_i/\rho^2)\delta_j^2}\, b_{0j}. \tag{23}$$

The remaining $M+1$ unknowns b_{0j} are determined by the $(M+1)$ boundary conditions at the inner sphere:

$$\big(\mu_i \tilde{S}_i(p,r) - \rho\, \partial_r \tilde{S}_i(p,r)\big)_{r=\rho} = 0 \quad (i=0,\dots,M) \tag{24}$$

that implies $(M+1)$ linear relations

$$\sum_{j=0}^{M} b_{ij} c_{ij} = a_i \mu_i \qquad (i=0,\dots,M), \tag{25}$$

where

$$\begin{aligned} c_{ij} &= \left(\rho\big(\partial_r v(\delta_j,r)\big)_{r=\rho} - \mu_i v(\delta_j,\rho)\right) \\ &= \sinh(\beta\delta_j)\big((1+\beta)\delta_j^2 - 1 - \mu_i\big) + \delta_j \cosh(\beta\delta_j)\big(\beta + \mu_i(1+\beta)\big). \end{aligned} \tag{26}$$

Substituting Eqs. (23) into these relations, one gets $M+1$ linear equations on the remaining $M+1$ unknowns b_{0j}:

$$\sum_{j=0}^{M} C_{ij} b_{0j} = a_i \mu_i \qquad (i=0,\dots,M), \tag{27}$$

with

$$C_{ij} = c_{ij} \times \begin{cases} 1 & (i=0), \\ \dfrac{k_{i0}}{p + k_i - (D_i/\rho^2)\delta_j^2} & (i>0). \end{cases} \tag{28}$$

Inverting the matrix C, one obtains b_{0j} and thus fully determines $\tilde{S}_i(p,r)$. Given that $\mu_i = 0$ for $i>0$, b_{0j} can formally be written as

$$b_{0j} = \frac{\mu\, f_{0j}(p)}{p\, f(p)}, \tag{29}$$

with

$$f(p) = \det(C), \qquad f_{ij}(p) = (-1)^{i+j} \mathscr{C}_{ij}, \tag{30}$$

where $\mathscr{C}_{ij}$ is the (i,j) minor of C, i.e., the determinant of the $M \times M$ matrix that results from deleting row i and column j of C. We get thus

$$\tilde{S}_0(p,r) = \frac{1}{p}\left(1 + \frac{w(p,r)}{f(p)}\right), \tag{31}$$

where

$$w(p,r) = \mu \sum_{j=0}^{M} f_{0j}(p)\, v(\delta_j,r). \tag{32}$$

This is the exact analytic solution of the problem in the Laplace domain. In order to get the solution in time domain, one needs to compute the poles of $\tilde{S}_0(p,r)$ which are given by zeros of the function $f(p)$ considered in the complex plane ($p \in \mathbb{C}$).

The survival probability $\tilde{S}_0(p,r)$ also determines the probability density of the first binding time, $\tilde{\psi}_1(p,r) = 1 - p\tilde{S}_0(p,r)$, from which

$$\tilde{\psi}_1(p,r) = -\frac{w(p,r)}{f(p)}. \tag{33}$$

In the general case $k_{i0} > 0$ (i.e., when buffers cannot bind calcium ions forever), one can show that $\tilde{\psi}_1(0,r) = 1$ that corresponds to the normalization of the probability density $\psi_1(t,r)$ (we omit the related asymptotic analysis of the minors $f_{ij}(p)$ and of $f(p)$ as $p \to 0$; see the example for one buffer in Sec. 4). As a consequence, $p=0$ is not a pole of $\tilde{S}_0(p,r)$, and $S_0(t,r)$ vanishes in the long time limit. In turn, if $k_{i0} = 0$ for some i, the calcium ions can be trapped forever by that buffer, and $\tilde{S}_0(t,r)$ reaches a

nonzero limit (the fraction of such trapped ions). In this specific case, $\tilde{\psi}_1(0,r) < 1$, i.e., the normalization of $\psi_1(t,r)$ does not hold. In practice, even if k_{i0} is very small, it is nonzero, and this pathologic situation does not occur. Note also that $\tilde{S}_0(p,r)$ determines the moments of the first binding times; in particular, the mean time is simply

$$\langle \mathscr{T} \rangle = \int_0^\infty dt\, t\, \psi_1(t,r) = \int_0^\infty dt\, S_0(t,r) = \tilde{S}_0(0,r), \tag{34}$$

where we integrated by parts and used that $\psi_1(t,r) = -\partial_t S_0(t,r)$ and $S_0(\infty,r) = 0$.

2.2 Occupancy probability

As discussed in the Methods Section, the probability density of the first binding times determines the occupancy probability $P(t,r)$ in the Laplace domain as

$$\tilde{P}(p,r) = \tilde{\psi}_1(p,r)\, \tilde{Q}(p), \tag{35}$$

where

$$\tilde{Q}(p) = \biggl(p + k_{\mathrm{off}}(1 - \tilde{\psi}(p,\rho))\biggr)^{-1}. \tag{36}$$

Substituting Eq. (33) into this equation yields

$$\tilde{Q}(p) = \biggl(p + k_{\mathrm{off}} + p k_{\mathrm{off}} \sum_{j=0}^{M} b_{0j}\, v(\delta_j,\rho)\biggr)^{-1}. \tag{37}$$

Next, substituting this expression and Eq. (75) into Eq. (35), we get explicitly

$$\tilde{P}(p,r) = -\frac{w(p,r)}{F(p)}, \tag{38}$$

with

$$F(p) = (p + k_{\mathrm{off}}) f(p) + k_{\mathrm{off}}\, w(p,\rho). \tag{39}$$

The poles of $\tilde{P}(p,r)$ are given by zeros of the function $F(p)$:

$$F(p_n) = 0 \qquad (n = 0,1,\ldots). \tag{40}$$

One can invert the Laplace transform by using the residue theorem. In particular, if the poles are simple, one gets

$$P(t,r) = \sum_{n=0}^{\infty} \bar{b}_n\, w(p_n,r) \exp(p_n t), \tag{41}$$

where

$$\bar{b}_n = -\frac{1}{\lim\limits_{p \to p_n} \partial_p F(p)}, \tag{42}$$

in which the derivative can be computed by using

$$\frac{\partial}{\partial p} v(\delta,r) = -\biggl(\cosh(\beta\delta) + \beta(1+\beta)\delta \sinh(\beta\delta)\biggr) \frac{\partial \delta}{\partial p} \tag{43}$$

and

$$\frac{\partial}{\partial p} c_{ij} = \frac{\partial \delta_j}{\partial p} \biggl\{ \cosh(\beta\delta_j)\bigl(\mu_i + \beta(1+\beta)\delta_j^2\bigr) + \delta_j \sinh(\beta\delta_j)\bigl(\mu_i\beta(1+\beta) + (\beta^2 + 2\beta + 2)\bigr) \biggr\}. \tag{44}$$

It can checked that $p_0 = 0$ whereas the other poles are strictly negative: $p_n < 0$. As a consequence, as $t \to \infty$, the probability $P(t,r)$ approaches a stationary value P_∞, which is independent of the starting point r and given by the residue at $p_0 = 0$. Summarizing these results, the occupancy probability takes the form

$$P(t,r) = P_\infty + \sum_{n=1}^{\infty} \exp(p_n t) \sum_{j=0}^{M} b_n^j \, v(\delta_j(p_n), r) \,, \tag{45}$$

where

$$b_n^j = \mu \, f_{0j}(p_n) \, \bar{b}_n. \tag{46}$$

Setting

$$\alpha_n = \rho \sqrt{-p_n/D_0}, \quad \alpha_n^{(j)} = -i\delta_j(p_n), \quad b_n^{(j)} = i b_n^j,$$

one can rewrite the occupancy probability in a more conventional form:

$$P(t,r) = P_\infty + \sum_{n=1}^{\infty} \exp\bigl(-\alpha_n^2 D_0 t/\rho^2\bigr) \sum_{j=0}^{M} b_n^{(j)} \, u(\alpha_n^{(j)}, r), \tag{47}$$

where

$$u(\delta, r) = \frac{\rho}{r} \biggl(\sin(\delta(R-r)/\rho) - (1+\beta)\delta \cos(\delta(R-r)/\rho) \biggr). \tag{48}$$

We note that the functions $\tilde{S}_0(p,r)$ and $\tilde{P}(p,r)$ involve complicated combinations of roots (e.g., square roots, see below) emerging from the zeros of the polynomial $H(z)$ in Eq. (22). As a consequence, the use of the residue theorem for evaluating the inverse Laplace transform of these functions is not straightforward as one needs to introduce cuts in the complex plane to properly deal with such multivariate functions. In addition, the application of Eq. (41) relies on the assumption of simple poles. In this paper, we do not provide rigorous mathematical analysis of both statements. In turn, we have checked the correctness and the accuracy of the derived formulas in time domain by comparison with the numerical inversion of the Laplace transform (not shown).

In summary, the analytic solution requires three numerical steps: (i) computation of δ_j^2 as the zeros of Eq. (22); (ii) inversion of the matrix C in Eq. (28), from which $f_{0j}(p)$, $f(p)$ and thus b_{0j} are found; and (iii) finding the zeros of $f(p)$ (for getting $S_0(t,r)$) or of $F(p)$ (for getting $P(p,r)$) for the inversion of the Laplace transform. We emphasize that δ_j and b_{0j} depend on p, i.e. one needs to perform the first two steps for all values of p at which $\tilde{S}_0(p,r)$ has to be found. In practice, the number of buffers, M, is not large so that these numerical steps can be done very rapidly and with any accuracy. We will discuss the cases $M = 0$ (Sec. 3) and $M = 1$ (Sec. 4), for which (some of) these steps can be done analytically.

2.3 Steady-state limit P_∞

As time t goes to infinity, the occupancy probability $P(t,r)$ from Eq. (47) approaches the steady-state limit P_∞, which is determined by the residue of $\tilde{P}(p,r)$ at the pole $p = 0$. Even though all the formulas determining $\tilde{P}(p,r)$ are given, the computation of this residue is technically involved, see the related analysis below for the particular cases of no buffer and one buffer. For this reason, we prefer to rely here on qualitative physical arguments that allow us to get the exact form of P_∞ without tedious computations.

In the steady-state, the system reaches an equilibrium between the free state, the buffer-bound states, and the sensor-bound state. Moreover, as the binding/unbinding kinetics on the sensor occurs only through the free state, one can separate the kinetics with the sensor and the kinetics with the buffers. The equilibrium kinetics with the sensor can be understood as a two-state switching model, governed by two exchange rates: k_{off} describes the transition from the sensor-bound state to the free state, whereas an effective rate $k_{0,s} = k_{\mathrm{on}}\, c_0$ characterizes the opposite transition, where c_0 is the equilibrated (homogeneous) concentration of calcium ions. If p_0 is the equilibrium fraction of calcium ions in the free state, then the conventional concentration (in M = mol/liter) reads $c_0 = p_0/(N_A V)$, where $V = 4\pi(R^3 - \rho^3)/3$ is the volume of the active zone. In this setting, the occupancy probability (i.e., the probability of finding the calcium ion bound to the sensor) is simply $P_\infty = k_{0,s}/(k_{0,s} + k_{\mathrm{off}})$ or, equivalently,

$$P_\infty = \frac{1}{1 + k_{\mathrm{off}} \frac{N_A V}{k_{\mathrm{on}}\, p_0}} \,. \tag{49}$$

The fraction p_0 of calcium ions in the free state can be determined from the equilibrium between the free state and buffer-bound states. For this purpose, we only consider the dynamics of the $(M+1)$-state switching model governed by the transition matrix W from Eq. (20) with $\gamma_i = k_i$ (i.e., at $p=0$). The steady-state distribution is determined by the eigenvector of $W^\dagger$ that corresponds to the eigenvalue 0: $(1, k_{01}/k_{10}, k_{02}/k_{20}, \ldots, k_{0M}/k_{M0})^\dagger$. After normalization to 1, the probability of finding the calcium ion in the free state (i.e., the fraction of calcium ions in this state) is

$$p_0 = \left(1 + \sum_{j=1}^{M} \frac{k_{0j}}{k_{j0}}\right)^{-1}. \tag{50}$$

We get therefore

$$P_\infty = \left(1 + k_{\rm off} \frac{N_A V}{k_{\rm on}} \left(1 + \sum_{j=1}^{M} \frac{k_{0j}}{k_{j0}}\right)\right)^{-1}. \tag{51}$$

The same expression for P_∞ is retrieved for cases $M=0$ (Sec. 3) and $M=1$ (Sec. 4) from the rigorous computation of the residue.

3 No buffer case

The survival probability for no buffer case is well known (see[9] and references therein). For illustrative purposes, we retrieve this survival probability from our general approach. This step is also needed for finding the occupancy probability $P(t,r)$.

When there is no buffer ($M=0$), Eq. (22) is reduced to $H = \gamma_0 = p - (D_0/\rho^2)\delta^2 = 0$, from which $\delta_0 = \rho\sqrt{p/D_0}$. The matrix C consists of a single element $C_{00} = c_{00}$, from which $f_{00}(p) = 1$, $f(p) = c_{00}$, and thus $b_{00} = \mu/(pc_{00})$. The Laplace-transformed survival probability becomes then

$$\tilde{S}_0(p,r) = \frac{1}{p} + \frac{\mu\, v(\delta_0, r)}{p\, c_{00}(p)}, \tag{52}$$

where $c_{00}(p)$ is given by Eq. (26). Setting $\delta_0 = i\hat{\alpha}$, one can rewrite the equation $f(p) = 0$ on the poles of $\tilde{S}_0(p,r)$ in a trigonometric form

$$\sin(\hat{\alpha}\beta) = \frac{(\beta + (1+\beta)\mu)\,\hat{\alpha}}{1 + \mu + (1+\beta)\hat{\alpha}^2} \cos(\hat{\alpha}\beta), \tag{53}$$

which has infinitely many nonnegative solutions denoted as $\hat{\alpha}_n$, enumerated by $n = 0, 1, 2, \ldots$ (we use hat symbol here to distinguish the quantities determining $S_0(t,r)$ from similar quantities determining $P(t,r)$ below). The poles are $\hat{p}_n = -D_0\hat{\alpha}_n^2/\rho^2$. Note that the pole corresponding to $\hat{\alpha}_0 = 0$ provides the contribution $-1/p$ that precisely compensates the term $a_0 = 1/p$, and thus it will be excluded. The inverse Laplace transform is then obtained by the residue theorem:

$$S_0(t,r) = \sum_{n=1}^{\infty} \hat{b}_n\, u(\hat{\alpha}_n, r) \exp\bigl(-\hat{\alpha}_n^2 D_0 t/\rho^2\bigr), \tag{54}$$

where

$$\hat{b}_n = \frac{2\mu}{\hat{\alpha}_n} \left(\cos(\hat{\alpha}_n\beta)\bigl[\mu - \beta(\beta+1)\hat{\alpha}_n^2\bigr] - \hat{\alpha}_n \sin(\hat{\alpha}_n\beta)\bigl[\beta(\beta+1)\mu + (\beta^2 + 2\beta + 2)\bigr]\right)^{-1} \tag{55}$$

and $u(\delta, r)$ is given by Eq. (48). The derivative with respect to t yields the probability density of the first-binding time

$$\psi_1(t,r) = \frac{D_0}{\rho^2} \sum_{n=1}^{\infty} \hat{\alpha}_n^2\, \hat{b}_n\, u(\hat{\alpha}_n, r) \exp\bigl(-\hat{\alpha}_n^2 D_0 t/\rho^2\bigr). \tag{56}$$

Occupancy probability $P(t,r)$

The computation of the probability $P(t,r)$ follows the same lines. Setting $\delta_0 = i\alpha$ in Eq. (39), one gets

$$F = \frac{iD_0}{\rho^2} \left\{ \sin(\alpha\beta)\Bigl((\alpha^2 - \lambda)((1+\beta)\alpha^2 + 1 + \mu) + \lambda\mu\Bigr) - \alpha\cos(\alpha\beta)\Bigl((\alpha^2 - \lambda)(\beta + \mu(1+\beta)) + \lambda\mu(1+\beta)\Bigr) \right\}, \tag{57}$$

from which the equation on α reads

$$\sin(\alpha\beta) = \frac{\left[\alpha^2(\beta+\mu(1+\beta)) - \lambda\beta\right]\alpha\cos(\alpha\beta)}{\alpha^4(1+\beta) + \alpha^2(1+\mu-\lambda(1+\beta)) - \lambda}, \tag{58}$$

where $\lambda = k_{\rm off}\rho^2/D_0$. This equation has infinitely many positive zeros that we denote as α_n, with $n = 1,2,\ldots$ (the zero $\alpha_0 = 0$ will be considered separately). These zeros determine the poles: $p_n = -D_0\alpha_n^2/\rho^2$. Since $w(p_n,r) = i\mu u_{\alpha_n}(r)$ with $u(\delta,r)$ given by Eq. (48), we obtain by the residue theorem

$$P(t,r) = \frac{1}{1+\lambda\frac{(\beta+1)^3-1}{3\mu}} + \sum_{n=1}^{\infty} b_n\, u(\alpha_n,r)\, e^{-\alpha_n^2 D_0 t/\rho^2}, \tag{59}$$

where the first term comes from the residue at $p = 0$, and

$$b_n = -\frac{i\mu}{\lim\limits_{p\to p_n}\left(\partial_p F(p)\right)} = \frac{i\mu}{\frac{\rho^2}{2D_0\alpha_n}\left(\partial_\alpha F(\alpha)\right)_{\alpha=\alpha_n}}. \tag{60}$$

Recalling the definition of dimensionless parameters λ, μ and β, one easily checks that the first term in Eq. (59) coincides with the steady-state limit P_∞ in Eq. (51).

Taking the derivative of Eq. (57) with respect to α, one gets an explicit formula for b_n:

$$b_n = \frac{2\mu}{\sin(\alpha_n\beta)\left(\alpha_n^2 w_1 + w_2\right) + \alpha_n\cos(\alpha_n\beta)\left(\alpha_n^2 w_3 + w_4\right)}, \tag{61}$$

with

$$\begin{aligned}
w_1 &= 4(1+\beta) + \beta(\beta+\mu(1+\beta)),\\
w_2 &= 2(1+\mu-\lambda(1+\beta)) - \lambda\beta^2,\\
w_3 &= \beta(1+\beta),\\
w_4 &= \beta(1+\mu-\lambda(1+\beta)) - 3(\beta+\mu(1+\beta)).
\end{aligned} \tag{62}$$

Limiting cases

In the limit $k_{\rm off} = 0$ (or $\lambda = 0$), there is no desorption event, and $\tilde{Q}(p) = 1/p$ according to Eq. (36). In this case,

$$\tilde{P}(p,r) = \frac{\tilde{\psi}_1(p,r)}{p} = \frac{1-\tilde{S}_0(p,r)}{p},$$

and thus $P(t,r) = 1 - S_0(t,r)$, as expected. One can also check that the solutions α_n coincide with $\hat{\alpha}_n$.

In turn, in the limit of perfectly adsorbing sensor (i.e., with infinitely fast binding kinetics: $k_{\rm on} = \mu = \infty$), Eq. (53) is reduced to

$$\sin(\hat{\alpha}_n\beta) = (1+\beta)\hat{\alpha}_n\cos(\hat{\alpha}_n\beta), \tag{63}$$

and the survival probability becomes

$$S_0(t,r) = \frac{2\rho}{r}\sum_{n=1}^{\infty}\exp\left(-\hat{\alpha}_n^2 D_0 t/\rho^2\right) \times \frac{\sin\left(\hat{\alpha}_n\frac{R-r}{\rho}\right) - (1+\beta)\hat{\alpha}_n\cos\left(\hat{\alpha}_n\frac{R-r}{\rho}\right)}{\hat{\alpha}_n\left(\cos(\hat{\alpha}_n\beta) - \beta(1+\beta)\hat{\alpha}_n\sin(\hat{\alpha}_n\beta)\right)}. \tag{64}$$

The probability density $\psi_1(t,r) = -\partial_t S_0(t,r)$ is obtained by taking the derivative with respect to t. Note that in this limit, the unbinding events are effectively suppressed as a particle that unbinds from such a sensor immediately re-binds. As a consequence, one gets again $P(t,r) = 1 - S_0(t,r)$.

Other results

Mean first-binding time

The mean first-binding time reads

$$\langle\mathcal{T}\rangle_r = \tilde{S}_0(0,r) = \frac{2r\rho(R^3-\rho^3)/\mu + 2\rho R^3(r-\rho) - r\rho^2(r^2-\rho^2)}{6r\rho^2 D_0}, \tag{65}$$

while the mean excursion time (at $r=\rho$) is

$$\langle \mathscr{T} \rangle_\rho = \frac{R^3-\rho^3}{3D_0\rho\mu} = \frac{\rho V}{\mu D_0 A} \,. \tag{66}$$

where V is the volume of the domain Ω_0 and A is the area of the sensor. As a consequence, the mean first-binding time, which is essentially proportional to the volume of the active zone, is a useless characteristics in this situation. In turn, the mode (i.e., the position of the density maximum, i.e., the most probable value) is representative.

Asymptotic analysis of the smallest eigenvalue

The long-time behavior of $\psi_1(t,r)$, $Q(t)$, and the probability $P(t,r)$, is determined by the smallest absolute value of the pole $|p_1|$ of the underlying Laplace-transformed quantity. Let us first consider the density $\psi_1(t,r)$, for which the smallest $|p_1|$ is determined by $\hat{\alpha}_1$. Denoting $x=\hat{\alpha}_1\beta$ and assuming that $x\to 0$, one can use the Taylor expansion of Eq. (53) to determine the asymptotic behavior of $\hat{\alpha}_1$ for large β. In the lowest order in $1/\beta$, we get

$$\hat{\alpha}_1^2 \simeq \frac{3\mu}{(1+\mu)\beta^3} \simeq \frac{3\mu\rho^3}{(1+\mu)R^3} \,. \tag{67}$$

According to Eq. (54), the above relation determines the slowest decay rate of the survival probability, $\rho^2/(D_0\hat{\alpha}_1^2)$, which is close to the mean time (66) when $\rho \ll R$.

Short-time asymptotic behavior

The short-time asymptotic behavior corresponds to the limit $p\to\infty$. In this limit, Eq. (38) becomes in the leading order in $1/p$:

$$\tilde{P}(p,r) \simeq \frac{\mu\sqrt{D_0}\,\exp\bigl(-(r-\rho)\sqrt{p/D_0}\bigr)}{r\,p^{3/2}} \,, \tag{68}$$

from which the short-time asymptotic behavior follows for $r>\rho$

$$P(t,r) \simeq \frac{4(D_0t)^{3/2}\mu}{\sqrt{\pi}r(r-\rho)^2}\,\exp\bigl(-(r-\rho)^2/(4D_0t)\bigr). \tag{69}$$

This asymptotic behavior is applicable at times as short as $t \ll (r-\rho)^2/(4D_0)$. In turn, for $r=\rho$, Eq. (68) yields

$$P(t,\rho) \simeq \frac{2\sqrt{D_0}\mu}{\sqrt{\pi}\rho}\,t^{1/2} \quad (t\to 0)\,. \tag{70}$$

4 One buffer case

For a single buffer ($M=1$), Eq. (22) reads

$$H = \bigl(p+k_{01}-(D_0/\rho^2)z\bigr)\bigl(p+k_{10}-(D_1/\rho^2)z\bigr) - k_{01}k_{10}, \tag{71}$$

and its two zeros determine δ_0 and δ_1:

$$\delta_0^2 = \frac{\rho^2}{2D_0D_1}\left(D_0(p+k_{10})+D_1(p+k_{01}) - \sqrt{(D_0(p+k_{10})-D_1(p+k_{01}))^2+4D_0D_1k_{01}k_{10}}\right), \tag{72}$$

$$\delta_1^2 = \frac{\rho^2}{2D_0D_1}\left(D_0(p+k_{10})+D_1(p+k_{01}) + \sqrt{(D_0(p+k_{10})-D_1(p+k_{01}))^2+4D_0D_1k_{01}k_{10}}\right). \tag{73}$$

Getting

$$\begin{aligned} f(p) &= C_{00}C_{11}-C_{01}C_{10}, \\ f_{00}(p) &= C_{11}, \quad f_{01}(p) = -C_{10} \end{aligned} \tag{74}$$

from the 2×2 matrix C, one obtains the coefficients b_{0j}

$$\begin{aligned} b_{00} &= \frac{C_{11}\,\mu}{p(C_{00}C_{11}-C_{01}C_{10})}\,, \\ b_{01} &= -\frac{C_{10}\,\mu}{p(C_{00}C_{11}-C_{01}C_{10})}\,, \end{aligned} \tag{75}$$

where the elements C_{ij} are given explicitly by Eq. (28). We obtain thus

$$\tilde{S}_0(p,r) = \frac{1}{p} + b_{00}\, v(\delta_0,r) + b_{01}\, v(\delta_1,r). \tag{76}$$

In order to invert the Laplace transform, one needs to determine the poles of $\tilde{S}_0(p,r)$ that are given by the zeros $\hat{p}_n$ of the function $f(p)$. There are infinitely many zeros and they are nonpositive: $\hat{p}_n \le 0$. To compute the residues, one needs the derivative of $f(p)$ with respect to p, which can be evaluated by using Eq. (44) and

$$\frac{\partial \delta_j}{\partial p} = \frac{\rho^2}{2\delta_j} \frac{2p + k_{01} + k_{10} - (D_0 + D_1)\delta_j^2/\rho^2}{D_0(p+k_{10}) + D_1(p+k_{01}) - 2D_0D_1\delta_j^2/\rho^2}. \tag{77}$$

Finally, we proceed to check that the two zeros of $f(p)$, $p = 0$ and $p = -(k_{01} + k_{10})$, are not the poles of $\tilde{S}_0(p,r)$, and thus excluded from the analysis.

(i) In the limit $p \to 0$, we get

$$\delta_0^2 \simeq \rho^2 \frac{k_{01} + k_{10}}{D_0 k_{10} + D_1 k_{01}} p + O(p^2), \tag{78a}$$

$$\delta_1^2 \simeq \frac{\rho^2 (D_0 k_{10} + D_1 k_{01})}{D_0 D_1} + O(p), \tag{78b}$$

$$v(\delta_0,\rho) \simeq -\delta_0 - \delta_0^3(\beta^3/3 + \beta^2/2) + O(\delta_0^5), \tag{78c}$$

$$\begin{aligned} C_{00} &\simeq \mu\delta_0 + \delta_0^3\left(\frac{\beta^3}{3} + \beta^2 + \beta + \mu\left(\frac{\beta^3}{3} + \frac{\beta^2}{2}\right)\right) + O(\delta_0^5), \\ C_{10} &\simeq \delta_0^3\left(\frac{\beta^3}{3} + \beta^2 + \beta\right) + O(\delta_0^5), \end{aligned} \tag{78d}$$

whereas C_{01} and C_{11} approach constants. We get thus

$$\begin{aligned} b_{00} &\simeq \frac{\mu}{p} \frac{C_{11}}{\mu\delta_0 C_{11} - O(\delta_0^3)} = \frac{1}{\delta_0 p} + O(p^{-1/2}), \\ b_{01} &\simeq -\frac{\mu}{p} \frac{O(\delta_0^3)}{\mu\delta_0 C_{11} - O(\delta_0^3)} = O(1). \end{aligned} \tag{79}$$

Since $v(\delta_0,r) = -\delta_0 + O(\delta_0^3)$, the singularities from $a_0 = 1/p$ and $b_0 v(\delta_0,r)$ cancel each other so that $p = 0$ is not a pole of $\tilde{S}_0(p,r)$.

(ii) Setting $p = -(k_{01} + k_{10}) + \varepsilon$, one has

$$\begin{aligned} \delta_0^2 &= -\frac{D_0 k_{01} + D_1 k_{10}}{D_0 D_1} + O(\varepsilon), \\ \delta_1^2 &= \frac{k_{01} + k_{10}}{D_0 k_{01} + D_1 k_{10}} \varepsilon + O(\varepsilon^2). \end{aligned} \tag{80}$$

As a consequence, we get

$$C_{01} \simeq \mu\delta_1 = O(\varepsilon^{1/2}), \qquad C_{11} \simeq \delta_1^3 = O(\varepsilon^{3/2}), \tag{81}$$

whereas a_0, C_{00} and C_{10} approach constants. We obtain then

$$\begin{aligned} b_{00} &= \frac{\mu}{p} \frac{C_{11}}{C_{00}C_{11} - C_{01}C_{10}} = O(1), \\ b_{01} &= -\frac{\mu}{p} \frac{C_{10}}{C_{00}C_{11} - C_{01}C_{10}} \simeq \frac{\mu}{C_{01} p} \simeq \frac{1}{\delta_1 p} = O(\varepsilon^{-1/2}). \end{aligned} \tag{82}$$

Since $v(\delta_1,r) \simeq -\delta_1$, the term $b_{01} v(\delta_1,r)$ has no singularity so that $p = -(k_{01} + k_{10})$ is not a pole of $\tilde{S}_0(p,r)$.

We conclude that

$$S_0(t,r) = \sum_{n=1}^{\infty} \left(\hat{b}_n^0\, v\big(\delta_0(\hat{p}_n),r\big) + \hat{b}_n^1\, v\big(\delta_1(\hat{p}_n),r\big)\right) \exp(\hat{p}_n t), \tag{83}$$

where $v(\delta, r)$ is given by Eq. (14), and

$$\hat{b}_n^0 = \frac{\mu C_{11}(\hat{p}_n)}{\hat{p}_n f'(\hat{p}_n)}, \qquad \hat{b}_n^1 = -\frac{\mu C_{10}(\hat{p}_n)}{\hat{p}_n f'(\hat{p}_n)}. \tag{84}$$

The derivative with respect to t yields

$$\psi_1(t,r) = \sum_{n=1}^{\infty} \Big(\hat{b}_n^0 v\big(\delta_0(\hat{p}_n), r\big) + \hat{b}_n^1 v\big(\delta_1(\hat{p}_n), r\big) \Big) \times |\hat{p}_n| \exp(\hat{p}_n t). \tag{85}$$

Probability $P(t,r)$

Similarly, the inversion of $\tilde{P}(p,r)$ involves the zeros p_n of $F(p)$ from Eq. (39) that can be written explicitly as:

$$F(p) = (p + k_{\mathrm{off}}) f(p) + k_{\mathrm{off}} \mu \big(C_{11} v(\delta_0, \rho) - C_{10} v(\delta_1, \rho) \big), \tag{86}$$

with $f(p)$ from Eq. (74). As previously, one can show that the zero $p = -(k_{01} + k_{10})$ is not a pole of $\tilde{P}(p,r)$. In turn, $p = 0$ is a pole. In fact, using Eqs. (78), one get as $p \to 0$

$$F(p) \simeq \delta_0 p \left\{ \mu C_{11}(0) + \lambda \frac{D_0(k_{01}+k_{10})}{D_0 k_{10} + D_1 k_{01}} \left(\frac{\beta^3}{3} + \beta^2 + \beta \right) \times \big(C_{11}(0) - C_{01}(0) - \mu v(\delta_1(0), \rho) \big) \right\},$$

where $C_{01}(0)$, $C_{11}(0)$ and $\delta_1(0)$ denote the values of these functions evaluated at $p = 0$. In turn,

$$w(p,r) = \mu \big(C_{11} v(\delta_0, r) - C_{10} v(\delta_1, r) \big) \simeq -\mu C_{11}(0) \delta_0 + O(\delta_0^3),$$

so that the residue at $p = 0$ is

$$P_\infty = \left\{ 1 + \lambda \frac{D_0(k_{01}+k_{10})}{D_0 k_{10} + D_1 k_{01}} \frac{(1+\beta)^3 - 1}{3} \times \frac{C_{11}(0) - C_{01}(0) - \mu v(\delta_1(0), \rho)}{\mu C_{11}(0)} \right\}^{-1}, \tag{87}$$

which is independent of the starting point r. After simplifications, we have

$$P_\infty = \left(1 + \lambda \frac{(1+\beta)^3 - 1}{3\mu} (1 + k_{01}/k_{10}) \right)^{-1}. \tag{88}$$

This expression coincides with Eq. (51).

We get thus

$$P(t,r) = P_\infty + \sum_{n=1}^{\infty} \Big(b_n^0 v\big(\delta_0(p_n), r\big) + b_n^1 v\big(\delta_1(p_n), r\big) \Big) e^{p_n t}, \tag{89}$$

with

$$b_n^0 = -\frac{\mu C_{11}(p_n)}{F'(p_n)}, \qquad b_n^1 = \frac{\mu C_{10}(p_n)}{F'(p_n)}. \tag{90}$$

One fixed buffer

For the fixed buffer ($D_1 \to 0$), Eqs. (72) yield

$$\delta_0^2 = \frac{\rho^2}{D_0} \left(p + k_{01} - \frac{k_{01} k_{10}}{p + k_{10}} \right), \qquad \delta_1^2 \to \infty. \tag{91}$$

As a consequence, one needs to treat this case separately to avoid diverging terms.

The last relation in Eqs. (91) implies that

$$c_{i1} \simeq \frac{1}{2} (1+\beta) \delta_1^2 e^{\beta \delta_1} \to \infty \qquad (i = 0, 1).$$

In addition, we have

$$C_{00} = c_{00}, \qquad C_{10} = \frac{c_{10} k_{10}}{p + k_{10}}, \qquad C_{11} = \frac{c_{11} k_{10}}{p + k_{10}},$$

so that in the limit $D_1 \to 0$, we get

$$b_{00} = \frac{\mu}{p\,c_{00}}, \qquad b_{01} = 0, \tag{92}$$

given that

$$\frac{C_{01}}{C_{11}} = \frac{c_{01}}{c_{11}\frac{k_{10}}{p+k_{10}-(D_1/\rho^2)\delta_1^2}} = \frac{c_{01}}{c_{11}\frac{p+k_{10}-(D_0/\rho^2)\delta_1^2}{k_{01}}} \to 0. \tag{93}$$

We conclude that

$$\tilde{S}_0(p,r) = \frac{1}{p} + \frac{\mu\,v(\delta_0,r)}{p\,c_{00}}, \tag{94}$$

i.e., we retrieved the solution (52) for the case without buffer, in which $\delta_0 = \rho\sqrt{p/D_0}$ is replaced by $\delta_0 = \rho\sqrt{p'/D_0}$, where

$$p' = p + k_{01} - \frac{k_{01}k_{10}}{p+k_{10}}. \tag{95}$$

The fixed buffer is expected to slow down the arrival onto the sensor because of binding calcium ions and thus stopping their diffusion. In particular, one can notice this effect in an increase of the mean first-binding time to the sensor, given by $\tilde{S}_0(0,r)$. Noting that $p' = 0$ from Eq. (95) at $p = 0$, one finds that the mean first-binding time without buffer, $\langle \mathscr{T}_{nb} \rangle$, is multiplied by the factor $(1 + k_{01}/k_{10})$ in the presence of a fixed buffer:

$$\langle \mathscr{T} \rangle = \tilde{S}_0(0,r) = \langle \mathscr{T}_{nb} \rangle \left(1 + \frac{k_{01}}{k_{10}}\right). \tag{96}$$

The relation to the former solution without buffer allows one to easily invert the Laplace transform. In fact, the former poles of $\tilde{S}_0(p,r)$ were $\hat{p}_n = -D_0\hat{\alpha}_n^2/\rho^2$. Inverting the relation (95), one can see that each former pole $\hat{p}_n$ splits in two new poles $\hat{p}_{n,1} = -\lambda_{n,1}$ and $\hat{p}_{n,2} = -\lambda_{n,2}$, with

$$\lambda_{n,1} = \frac{\sigma_n - \sqrt{\sigma_n^2 - 4k_{10}D_0\hat{\alpha}_n^2/\rho^2}}{2}, \tag{97a}$$

$$\lambda_{n,2} = \frac{\sigma_n + \sqrt{\sigma_n^2 - 4k_{10}D_0\hat{\alpha}_n^2/\rho^2}}{2}, \tag{97b}$$

with $\sigma_n = D_0\hat{\alpha}_n^2/\rho^2 + k_{01} + k_{10}$. As a consequence, the inverse Laplace transform of Eq. (94) becomes

$$S_0(t,r) = \sum_{n=1}^{\infty} \hat{b}_n\,u(\hat{\alpha}_n,r)\left(c_{n,1}\,e^{-\lambda_{n,1}t} + c_{n,2}\,e^{-\lambda_{n,2}t}\right), \tag{98}$$

where $u(\delta,r)$ is given by Eq. (48), the coefficients $\hat{b}_n$ are given by Eq. (55), and the weights

$$c_{n,1} = \frac{D_0\hat{\alpha}_n^2/\rho^2}{\lambda_{n,1}}\,\frac{1}{1 + \frac{k_{01}k_{10}}{(\lambda_{n,1}-k_{10})^2}}, \tag{99a}$$

$$c_{n,2} = \frac{D_0\hat{\alpha}_n^2/\rho^2}{\lambda_{n,2}}\,\frac{1}{1 + \frac{k_{01}k_{10}}{(\lambda_{n,2}-k_{10})^2}}, \tag{99b}$$

appear from the change of variables: $dp'/dp = 1 + k_{01}k_{10}/(p+k_{10})^2$, see Eq. (95), and from the factor $1/p$ in the second term of Eq. (94). Note that if $k_{01} = k_{10} = 0$, one has $\lambda_{n,1} = 0$ and $\lambda_{n,2} = D_0\hat{\alpha}_n^2/\rho^2$, and one retrieves Eq. (54).

Note also that $\lambda_{n,1} \to k_{10}$ and $\lambda_{n,2} \to D_0\hat{\alpha}_n^2/\rho^2$ as $n \to \infty$ and thus $c_{n,1} \to 0$ and $c_{n,2} \to 1$. In other words, the exchange kinetics does not affect the high-frequency eigenmodes.

Substituting Eq. (98) into (35, 36), we get

$$\tilde{P}(p,r) = -\frac{\mu\,v(\delta_0,r)}{(p+k_{\rm off})c_{00} + k_{\rm off}\,\mu\,v(\delta_0,\rho)}, \tag{100}$$

so that one needs to find zeros of the denominator of this expression. As in the former case for $\tilde{S}_0(p,r)$, one can expect two sequences of zeros: $p_{n,1} \to -k_{10}$ and $p_{n,2} \to -\infty$. In fact, when $p \to -k_{10} + 0$, p' from Eq. (95) diverges to $-\infty$, so that there are infinitely many zeros accumulating towards $-k_{10}$. This accumulation requires a more subtle numerical procedure to calculate zeros.

5 Calcium channel model used for Monte Carlo simulations

We describe a VGCC by a 3-state Hodgkin and Huxley gating model[10] so that the calcium release was modeled according to:

$$C_0 \underset{\beta(V(t))}{\overset{2\alpha(V(t))}{\rightleftarrows}} C_1 \underset{2\beta(V(t))}{\overset{\alpha(V(t))}{\rightleftarrows}} O \overset{k(t)}{\rightarrow} Ca^{2+}, \tag{101}$$

with two closed states C_0, C_1 and one open state O of the VGCC. Here $\alpha(V(t))$ and $\beta(V(t))$ are voltage dependent rates, computed as

$$\begin{aligned} \alpha(V(t)) &= \exp(V(t)/20.5), \\ \beta(V(t)) &= 0.14\,\exp(-V(t)/15), \end{aligned} \tag{102}$$

for a given AP waveform $V(t)$ in mV. The dynamics starts from the close state C_0. The parameters in these rates were adjusted such that the resulting single channel open probability, current duration, and peak match experimentally observed quantities[11].

The calcium ions are released from the open channel with the rate:

$$k(t) = \frac{g}{2e}(V(t) - V_{\text{rev}}), \tag{103}$$

where $g = 3.3$ pS (picoSiemen) is the single channel conductance[12], e is the elementary charge, and $V_{\text{rev}} = -45$ mV is the reversal potential[11].

6 Supplementary Figures

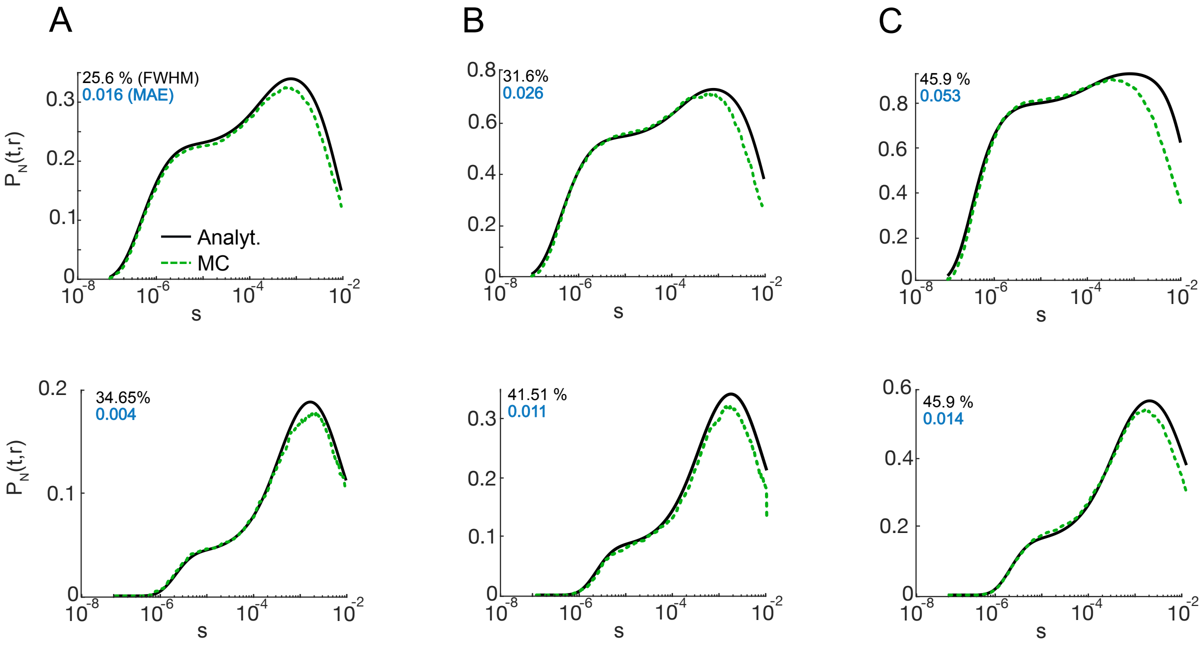

Supplementary Figure 1. $P(t,r)$ with slow unbinding kinetics ($k_{on} = 0.157$ mM^{-1}ms^{-1}) for 50 (**A**), 100 (**B**) and 200 (**C**) simultaneously released ions for CD of 15 nm (top row) and 45 nm (bottom row). Black and green lines show respectively analytical and MC results. The black and blue inset text on each plot represent FWHM error and MAE between analytical and MC results correspondingly.

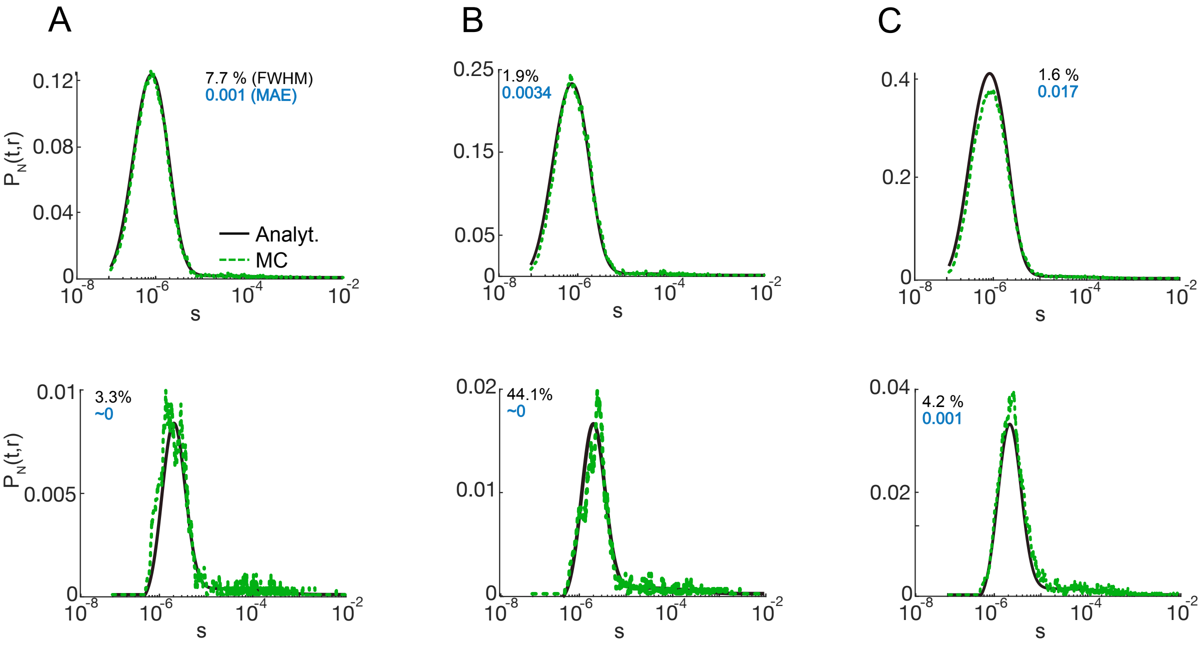

Supplementary Figure 2. $P(t,r)$ with fast unbinding kinetics ($k_{on} = 1570$ mM^{-1}ms^{-1}) for 50 (**A**), 100 (**B**) and 200 (**C**) simultaneously released ions for CD of 15 nm (top row) and 45 nm (bottom row). Black and green lines show respectively analytical and MC results. The black and blue inset text on each plot represent FWHM error and MAE between analytical and MC results correspondingly.

References

1. G. Yin and C. Zhu *Hybrid Switching Diffusions: Properties and Applications* (Springer, New York, 2010).
2. G. Yin and C. Zhu, "Properties of solutions of stochastic differential equations with continuous-state-dependent switching", J. Diff. Eq. **249**, 2409-2439 (2010).
3. N. A. Baran, G. Yin, and C. Zhu, "Feynman-Kac formula for switching diffusions: connections of systems of partial differential equations and stochastic differential equations", Adv. Diff. Eq. 2013:315 (2013).
4. D. S. Grebenkov, "Time-averaged mean square displacement for switching diffusion", Phys. Rev. E **99**, 032133 (2019).
5. M. Freidlin, *Functional Integration and Partial Differential Equations*, Annals of Mathematics Studies (Princeton University Press, Princeton, New Jersey, 1985).
6. D. S. Grebenkov, "Partially Reflected Brownian Motion: A Stochastic Approach to Transport Phenomena", in "Focus on Probability Theory", Ed. L. R. Velle, pp. 135-169 (Nova Science Publishers, 2006).
7. D. S. Grebenkov, "Residence times and other functionals of reflected Brownian motion", Phys. Rev. E **76**, 041139 (2007).
8. A. Singer, Z. Schuss, A. Osipov, and D. Holcman, "Partially Reflected Diffusion", SIAM J. Appl. Math. **68**, 844 (2008).
9. D. S. Grebenkov, R. Metzler, and G. Oshanin, "Strong defocusing of molecular reaction times results from an interplay of geometry and reaction control", Commun. Chem. **1**, 96 (2018).
10. A. L. Hodgkin and A. F. Huxley, "A quantitative description of membrane current and its application to conduction and excitation in nerve", J. Physiol. **117**, 500-544 (1952).
11. Y. Nakamura, H. Harada, N. Kamasawa, K. Matsui, J. S. Rothman, R. Shigemoto, R. A. Silver, D. A. DiGregorio, and T. Takahashi, "Nanoscale distribution of presynaptic Ca 2+ channels and its impact on vesicular release during development", Neuron **85**, 145-158 (2015).
12. J. Sheng, L. He, H. Zheng, L. Xue, F. Luo, W. Shin, T. Sun, T. Kuner, D. T. Yue, and L.-G. Wu, "Calcium-channel number critically influences synaptic strength and plasticity at the active zone", Nature Neurosci. **15**, 998-1006 (2012).
13. L.-Y. Wang, E. Neher, and H. Taschenberger, "Synaptic vesicles in mature calyx of Held synapses sense higher nanodomain calcium concentrations during action potential-evoked glutamate release", J. Neurosci. **28**, 14450-14458 (2008).
